# Dinuclear Copper(II) Complexes with Schiff Bases Derived from 2-Hydroxy-5-Methylisophthalaldehyde and Histamine or 2-(2-Aminoethyl)pyridine and Their Application as Magnetic and Fluorescent Materials in Thin Film Deposition

**DOI:** 10.3390/ijms21134587

**Published:** 2020-06-28

**Authors:** Magdalena Barwiolek, Anna Kaczmarek-Kędziera, Tadeusz M. Muziol, Dominika Jankowska, Julia Jezierska, Alina Bieńko

**Affiliations:** 1 Faculty of Chemistry, Nicolaus Copernicus University in Torun, 87-100 Torun, Poland; tadeuszmuziol@wp.pl (T.M.M.); 278998@stud.umk.pl (D.J.); 2 Faculty of Chemistry, University of Wroclaw, 14 Joliot-Curie, 50-383 Wroclaw, Poland; julia.jezierska@chem.uni.wroc.pl (J.J.); alina.bienko@uwr.edu.pl (A.B.)

**Keywords:** Schiff bases, dicopper complexes, magnetic properties, DFT, EPR, fluorescence, thin layer

## Abstract

Two Cu(II) complexes, **1** and **2**, with tridentate Schiff bases derived from 2-hydroxy-5-methylisophthalaldehyde and histamine **HL1** or 2-(2-aminoethyl)pyridine **HL2**, respectively, were obtained and characterized by X-ray crystallography, spectroscopic (UV-vis, fluorescence, IR, and EPR), magnetic, and thermal methods. Despite the fact that the chelate formed by the NNO ligand donors (C26-C25H_2_-C24H_2_-N23=C23H-C22-C19Ph(O1)-C2(Ph)-C3H=N3-C4H_2_-C5H_2_-C6 fragment) are identical, as well as the synthesis of Cu(II) complexes (Cu:L = 2:1 molar ratio) was performed in the same manner, the structures of the complexes differ significantly. The complex **1**, {[Cu_2_(L1)Cl_2_]_2_[CuCl_4_]}·2MeCN·2H_2_O, consists of [Cu_2_(L1)Cl_2_]^+^ units in which Cu(II) ions are bridged by the **HL1** ligand oxygen and each of these Cu(II) ions is connected with Cu(II) ions of the next dimeric unit via two bridging Cl^−^ ions to form a chain structure. In the dinuclear [Cu_2_(L2)Cl_3_]⋅0.5MeCN complex **2**, each Cu(II) is asymmetrically bridged by the ligand oxygen and chloride anions, whereas the remaining chloride anions are apically bound to Cu(II) cations. In contrast to the complex **1**, the square-pyramidal geometry of the both Cu(II) centers is strongly distorted. The magnetic study revealed that antiferromagnetic interactions in the complex **2** are much stronger than in the complex **1**, which was corresponded with magneto-structural examination. Thin layers of the studied Cu(II) complexes were deposited on Si(111) by the spin coating method and studied by scanning electron microscopy (SEM/EDS), atomic force microscopy (AFM), and fluorescence spectroscopy. The Cu(II) complexes and their thin layers exhibited fluorescence between 489–509 nm and 460–464 nm for the compounds and the layers, respectively. Additionally, DFT calculations were performed to explain the structures and electronic spectral properties of the ligands.

## 1. Introduction

Schiff base metal complexes are an attractive research field due to their industrial and biological applications or potential use in material science [1,2,3]. They have attracted the attention of coordination chemists and inspired them to design and synthesize mono or polynuclear complexes of transition metals [4]. Transition metal complexes with N,O- donor Schiff bases are remarkable owing to their ability to accept unusual configurations, structural lability and sensitivity to environment [5,6]. Significant effort was made to design, synthesize, and modify complexes due to their ability to influence molecular magnetism. Studying magnetic interactions between the central Cu(II) ions in multinuclear complexes is the main objective of magneto-structural investigations [7,8]. 

Dinuclear copper complexes are also models of active copper containing enzymes, e.g., hemocyanin or cytochrome c [9,10], and they are meaningful as precursors in the chemistry of supramolecular and discrete molecular high-nuclearity Cu(II) complexes [11,12,13,14]. The variety of organic ligands is essential in coordination chemistry as they influence the magnetic, spectroscopic properties, and geometry of complexes [12,13,14,15,16,17,18,19,20,21,22,23,24,25]. Different species were used as bridging ligands in copper complexes, i.e., cyanate, thiocyanate [4,23], phenoxo [24], chloride [11]. The studies of a series of phenoxo- and hydroxo-bridged dicopper(II) complexes obtained from *bis*(2-methylpyridyl)aminomethyl as complexing arms with different substituting groups, e.g., CH_3_ or OCH_3,_ demonstrated that a slight modification of the ligand topology may significantly affect the spectroscopic and redox properties of the complexes and may lead to developing of transient, mixed-valent Cu(II)-Cu(III) complexes [12].

In the series of Cu(II) complexes with the tridentate Schiff base 1-[(2-pyridin-2-yl-ethylimino)-methyl]-naphthalen-2-ol, the presence of the chloride or acetate ion in the Cu(II) coordination sphere imposed square planar geometry of the copper(II) ion with C.N. = 4, whereas NO_3_^−^ ion forced the distorted square pyramid geometry with C.N. = 5. The chloride ligand presence in the Cu(II) coordination sphere resulted also in the formation of a stronger hydrogen bond network and possible existence of a Cu···Cl···Cu magnetic exchange pathway [5]. Moreover, the type of an anion coordinated with the Cu(II) ion significantly influenced the fluorescence of the complexes. The complexes exhibited blue luminescence in a solution and the lowest fluorescence intensity in the case of the Cu(II) compound with the nitrate ion in the coordination sphere was noted. Thin films of Cu(II) complexes obtained by the spin coating method exhibited fluorescence connected with the *d-d* transitions in the range of 490–550 nm.

The ligands derived from 2-hydroxy-5-methylisophthaldehyde were applied in the bi- and multinuclear copper(II) compounds synthesis [25,26,27]. It has been proved that this aldehyde plays an important role in the coordination chemistry because it functions as building blocks for binucleating macrocyclic and non-macrocyclic ligands. Chloro-bridged binuclear copper(II) complexes exhibit diverse structural properties and are applicable in molecular magnetism [27,28]. As a consequence, for these chloro-bridged complexes, several magnetic interaction pathways exist and both ferro- and antiferro-magnetism are possible depending on the nature of a coordinated ligand and distortions of the metal center geometry. Therefore, each type of a structural dimer has to be studied separately in order to draw magneto-structural correlations and determine the extent of this correlation.

The transition metal complexes constitute another interesting class of compounds, owing to their luminescent abilities and potential applications in organic optoelectronics [29,30]. Thin films of transition metal complexes with Schiff bases were obtained using wet or gaseous methods, e.g., chemical vapor deposition, cathode-luminescence or spin and dip coating. The optical properties of these materials were also studied and some of them exhibited fluorescence [5,31,32]. There are only a few reports on copper(II) compound materials. In the case of CuPc (Pc–tetra-tricarbethoxyethyl substituted phthalocyanine), the absorption increase was noted at higher copper concentration in the layers [30]. Films of Cu(II) complexes with Schiff bases derived from different aldehydes and (1*R*,2*R*)-(−)-cyclohexanediamine [32] or 2-(2-aminoethyl)pyridine [33] obtained with spin coating were homogenous, with compounds evenly spread over the silicon substrates, and exhibited fluorescence between 500–536 nm. The highest fluorescence intensity was observed for smooth layers which revealed the highest copper content.

There are still many questions regarding the fluorescence properties of compounds and thin films. The appropriate methods of establishing new materials should also be developed. New films can improve the key parameters such as high luminescence, thin designs, magnetic properties and provide new unique characteristics of the new devices such as smartphones, OLEDS or satellite navigation systems. Consequently, new compounds with fluorescent and magnetic properties that could be used in thin films are still sought for.

Therefore, we report the synthesis, structure, magnetic and spectroscopic properties of two binuclear Cu(II) complexes with Schiff bases derived from 2-hydroxy-5-methylisophthalaldehyde and histamine (**1**) or 2-(2-aminoethyl)pyridine (**2**). Additionally, the role of organic ligands in the structure formation of Cu(II) Schiff bases complexes and their influence on the strength of magnetic interaction were investigated. DFT calculations have been carried out to support the interpretation of the results of studying the optical properties of the ligands. The new copper(II) complexes were used as precursors of thin layers in the spin coating technique. The morphology of the layers was analyzed by AFM and SEM microscopy, and the fluorescence properties of the layers were also studied.

## 2. Results and Discussion

### 2.1. Ligand Synthesis and Characterization

**Figure 1:**
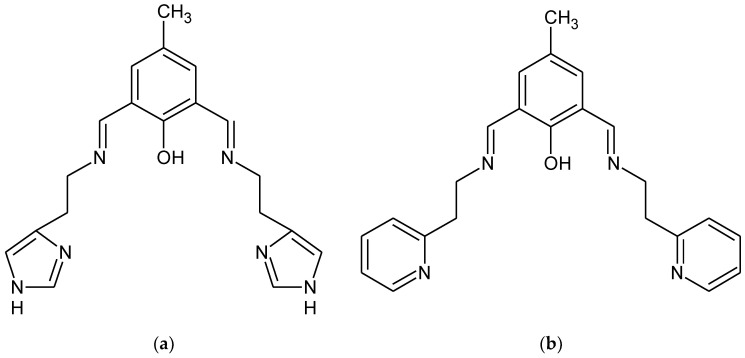
Chemical structures of (**a**) **HL1** and (**b**) **HL2**.

The 1:1 reaction of the corresponding aldehyde and primary amine forms the **HL1** and **HL2** ligands in yields ranging between 94.0% and 96.0% (Figure 1). The ligands were characterized by ^1^H, ^13^C, ^1^H ^13^C hmqc and hmbc NMR, UV-vis, IR and the elemental analysis. ^1^H, ^13^C NMR spectra are presented in Supplementary Figures S1–S4 Additionally, a theoretical chemical shift for ^1^H, ^13^C and ^15^N NMR for the **HL1** ligand and the **HL2** ligand [ppm] were also calculated with B3LYP/def2-TZVP approach in the gas phase and solvents (Supplementary Table S1). The results of the NMR analysis of the **HL2** ligand are similar to that observed previously [34]. The ligands are stable in air and soluble in several solutions such as chloroform, methanol, and acetonitrile. The IR spectra displayed in Supplementary Figures S5 and S6 exhibit peaks at 1635 and 1672 cm^−1^ from stretching vibrations of the azomethine group. The peaks at 3372, 3359 cm^−1^ are attributed to OH group vibrations [4]. Moreover, bands from aromatic rings stretching vibrations in the range of 1476–1372 cm^−1^ were noted.

### 2.2. Dinuclear Copper(II) Complexes: Synthesis and Characterization

Reactions of ligands with copper(II) chloride hydrate resulted in dinuclear copper(II) complexes formation. IR spectra of the compounds are displayed in Supplementary Figures S7 and S8. Upon complexation, the characteristic -C=N- stretching absorption band is shifted towards lower energies (1641–1609 cm^−1^). Another absorption band which is sensitive to ligand coordination originates from (Ph)C-O vibrations appearing in the range of 1252–1280 cm^−1^. The elemental analysis and X-ray studies confirmed the purity of the obtained compounds. The thermal stability of **1** and **2** complexes was studied by the thermogravimetric analysis from ambient temperature to 1000 °C under air. The final decomposition product was a mixture of copper and copper oxide (Supplementary Figures S9 and S10).

### 2.3. Structure Description

{[Cu_2_(L1)Cl_2_]_2_[CuCl_4_]}·2MeCN·2H_2_O (**1**)

**Figure 2:**
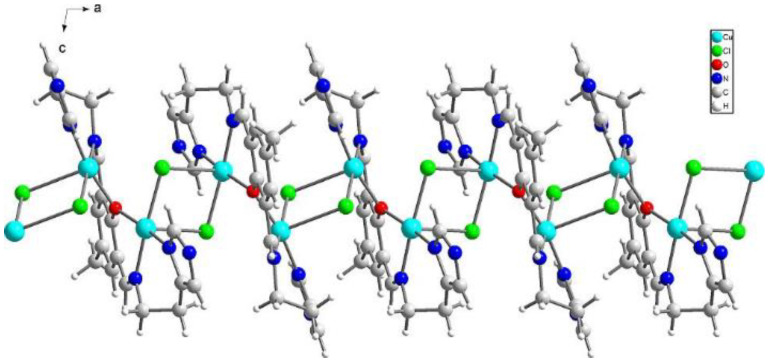
Perspective view of **1** along the crystallographic *b* axis.

{[Cu_2_(L1)Cl_2_]_2_[CuCl_4_]}·2MeCN·2H_2_O (**1**) crystallizes in triclinic *P*-1 space groups with the Cu3 atom positioned at the inversion center and all the remaining atoms found in the general positions. The structure consists of [Cu_2_(L1)Cl_2_]^+^ dimers with O1 oxygen atoms from the Schiff base connecting both of the copper ions. Subsequently, chloride anions couple those units into chains following the pattern: Cl1--[Cu1-O1-Cu2--Cl2--Cu2-O1-Cu1--Cl1]--Cu1-O1-Cu2--Cl2. Additionally, the structure is completed with [CuCl_4_]^2−^ anions as well as acetonitrile and water molecules (Figure 2).

**Figure 3:**
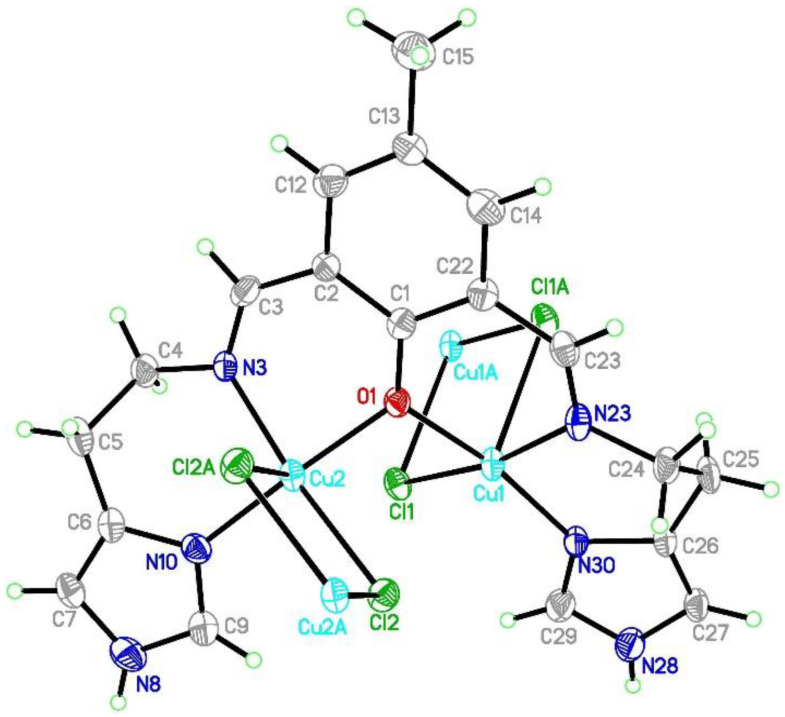
Asymmetric unit of the crystal structure of {[Cu_2_(L1)Cl_2_]_2_[CuCl_4_]}·2MeCN·2H_2_O (**1**). Ellipsoids are plotted at 30% probability level.

The Cu3 atom coordination sphere in CuCl_4_^2−^ anions consists of four chloride anions creating square-planar environment with Cu-Cl distances of 2.274(3) Å and angles of 89.63(10), 90.37(10), and 180° (Table 1). Cu1 and Cu2 atoms in [Cu_2_(L1)Cl_4_]^−^ are found in square pyramidal environment (τ = 0.003 and 0.058, respectively) with copper atoms shifted by 0.01 and 0.06 Å towards apical chloride anion (Figure 3 and Figures S11–S13).

The both basal planes composed of O1 oxygen atom, two nitrogen atoms, and one chloride anion form an angle of 58.20(12)°. In the coordination sphere of the Cu1 atom, the Cu-O/N bond lengths range from 1.956(5) to 2.025(7) Å, and for the Cu1-Cl1 bond, the length is 2.350(2) Å, whereas the apical Cl1[−*x*, −*y* + 1, −*z* + 2] chloride anions forms strongly elongated (2.849(2) Å) bonds. For the Cu2 atom, the Cu-O/N bonds range from 1.956(5) to 2.006(6) Å. Both of the chloride anions are bound to Cu2 at the distances of 2.344(2) and 2.786(2) Å revealing similar elongation to that observed for the Cu1 atom. Hence, for the both coordination spheres, the shortest distances are observed for O1 interacting symmetrically with two copper atoms. Cu1 and Cu2 atoms are found on the sides opposite to the central C11 plane. Both imidazole rings are twisted by 15.5(4)° and form angles of 9.1(3) and 12.7(2)° with the central C1 ring. The elongation of the apical Cu-Cl bonds is related to Jahn-Teller effect and they are similar to distances found for the chain structure reported by Bieńko et al. [35]. On the contrary, in [Cu_2_(L–O)(μ-Cl)_2_][Cu_2_Cl_6_]_0.5_ (L–O = 2,6-bis-[*N*-(2-pyridylethyl)- formidoyl]-4-methyl phenol) with very similar ligand a chain formation is not observed [36]. Copper is also found in square pyramidal environment in which two distinct Cu-Cl bonds are 2.324(2) and 2.802(2) as well as 2.689(2) and 2.335(2) Å long for Cl1 and Cl2 anions, respectively. The apical position is occupied also by bridging chloride anion but, in this case, chloride bridges are formed between the both copper(II) cations bound with the same organic ligand. In **1**, we do not observe such bridging; Cu1⋅⋅⋅Cl2 and Cu2⋅⋅⋅Cl1 distances are 2.984 and 2.885 Å, respectively and are beyond the bonding criterion defined in PLATON [37]. In **1**, the long apical bonds enabled chain formation running along *a* axis, but the chloride bridge is composed of two Cu-Cl bonds significantly differing in their length. There are three short intermetallic distances, 3.161, 3.787 and 3.727 Å, corresponding to Cu1⋅⋅⋅Cu2, Cu1⋅⋅⋅Cu1 and Cu2⋅⋅⋅Cu2 distances in the chain. In packing, we observe alternately arranged layers of cations and anions with water molecules positioned in the cationic sublattice; additionally, acetonitrile is found in the anionic network. The chain consists of dimers connected by asymmetric chloride bridges. They form *ab* layers due to π-π interactions between N30 and C1 rings and C15-H15B⋅⋅⋅N30 ring interactions. The crystal network is completed by contacts among different planes assured by C27-H27⋅⋅⋅Cl4[−*x*, −*y*, 1 − *z*] and N8-H8⋅⋅⋅Cl4[1 − *x*, −*y*, 2 − *z*] hydrogen bonds connecting dimer with [CuCl_4_]^2−^ block. The crystallization water molecule is also involved in hydrogen bond to the dimer: C29-H29⋅⋅⋅O34[−*x*, −*y*, 2 − *z*].

[Cu_2_(L2)Cl_3_]0.5MeCN (**2**)

**Figure 4:**
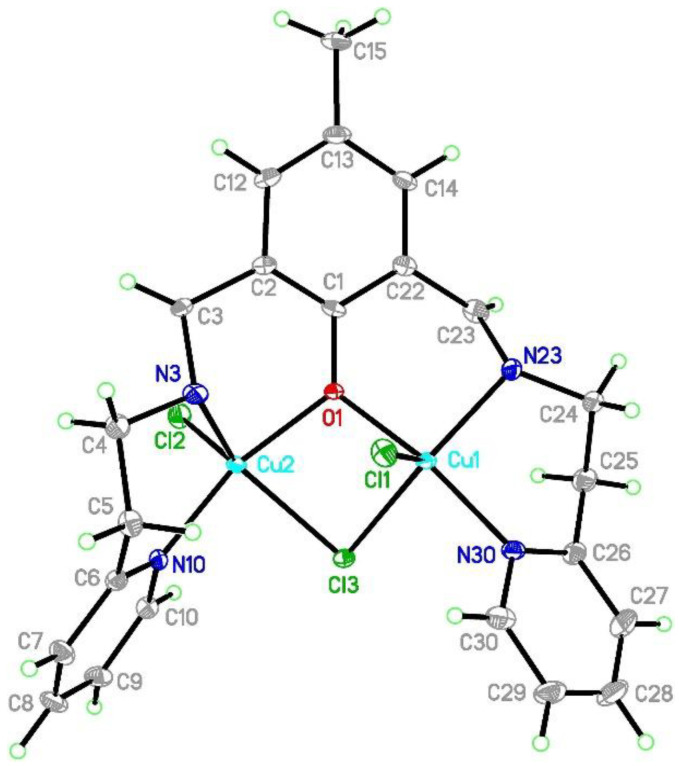
Asymmetric unit of [CuCl_3_(L2)]⋅0.5MeCN **2** with the thermal ellipsoids at 50% probability for clarity of the picture. The partially occupied acetonitrile molecule is omitted.

[Cu_2_Cl_3_(L2)]⋅0.5MeCN (**2**) crystallizes in the monoclinic *C*2 space group with Flack parameter at 0.007(2) which indicates that the absolute structure is correct and the pure enantiomer was obtained. In the asymmetric unit, the whole molecule derived from the above formula was found. The coordination sphere of the both copper(II) is five-coordinated and is composed of three chloride anions, one oxygen, and two nitrogen atoms (Figure 4). The copper coordination geometry is strongly distorted with τ_5_ parameters [38,39] of 0.413 and 0.391 for Cu1 and Cu2 centers, respectively. For the purposes of the further discussion, we assume that there are significantly deformed square pyramids with the shared edge and the non-bridging Cl1 and Cl2 in the apical positions at 2.4254(9) and 2.3906(9) Å for Cu1 and Cu2, respectively (Table 1). The common edge is formed by the O1 oxygen atom forming the shortest Cu-ligand bonds (1.954(2) for Cu1 and 1.982(2) Å for Cu2) and the bridging Cl3 anion (2.3926(8) Å for Cu1 and 2.4279(8) Å for Cu2). This chloride forms an asymmetric bridge with Cu-Cl3 distances differing by 0.03 Å. The sphere is completed by two nitrogen atoms: one from the pyridine ring and the other from the azomethine group. The latter forms significantly longer Cu-N bonds. The inspection of the bond lengths shows that Cu-N/O bonds are much more similar in the Cu2 coordination sphere than in the Cu1 one. The valence angles fall into two broad ranges, 80.32(7)–110.23(3) and 142.27(9)–167.08(11)° for Cu1, and 78.91(7)–109.63(9) and 139.87(9)–163.35(10)° for Cu2.

**Figure 5:**
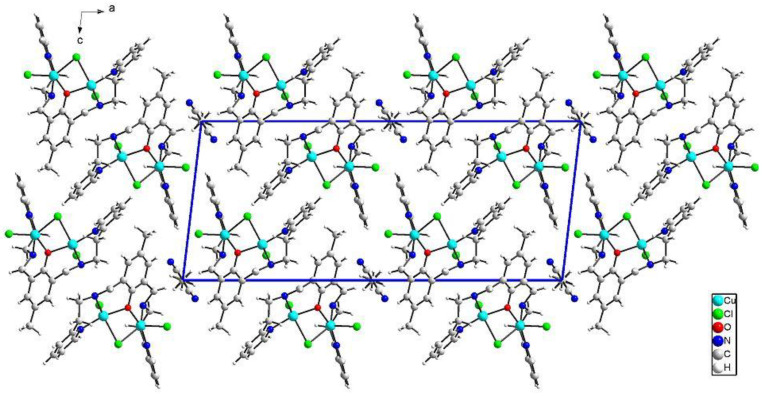
Packing of **2** along *b* axis reveals motif of π-π interactions between aromatic rings: stacking between two N10 units and rather edge-to-face in case of N30-N30 and N10-N30 interactions.

The structure stability is certainly increased by the formation of four non-planar six-membered chelate rings which assure some conformational flexibility. It can be expressed by torsion angles which show the most distinct values for C3-N3-C4-C5 (175.0(3)°) vs. C23-N23-C24-C25 (−94.8(3)°) and C1-C2-C3-N3 (30.8(5)°) vs. C1-C22-C23-N23 (−59.4(4)°). It results in different mutual orientations of the aromatic rings at 53.69(16)° (C1 phenyl/N10 pyridine), 35.22(17)° (C1 phenyl/N10 pyridine) and 63.98(16)° (N10 pyridine/N30 pyridine) and interactions formed in the crystal network. These torsion angles values show that the structure is not in an extended conformation, but the angle between basal planes of square pyramids for Cu1 and Cu2 is 48.1° and the Cl1-Cu1-Cu2-Cl2 torsion angle is 150.98° which results in separation between Cu(II) ions at 3.094 Å. The mutual orientation of the copper d orbitals should also affect magnetic properties. The crystal network is maintained via a few C-H⋅⋅⋅Cl hydrogen bonds and π-π interactions (Figure 5). The conformational flexibility of the linkers between the phenyl ring and the both pyridine rings assure stacking interactions between the two N10 pyridine moieties whereas the two N30 rings are tilted by 23.0°. This motif of the rings interacting along *a* axis is completed by the edge-to-face contact between the N10 and the N30 pyridines. Due to the torsional angles, the C1 phenyl ring also creates the edge-to-face π-π interactions only with the N30 ring. Every dimer forms two C-H⋅⋅⋅Cl hydrogen bonds (C5-H5B⋅⋅⋅Cl2[*x*, −1 + *y*, *z*] and C23-H23⋅⋅⋅Cl1[*x*, 1 + *y*, *z*]) with adjacent units translated along the *b* axis. The structure is completed by a partially occupied (0.5) acetonitrile molecule interacting via the C31-H31⋅⋅⋅C1[1 − *x*, −1 + *y*, 2 − *z*] ring.

### 2.4. DC Magnetic Measurements and EPR Spectra

**Figure 6:**
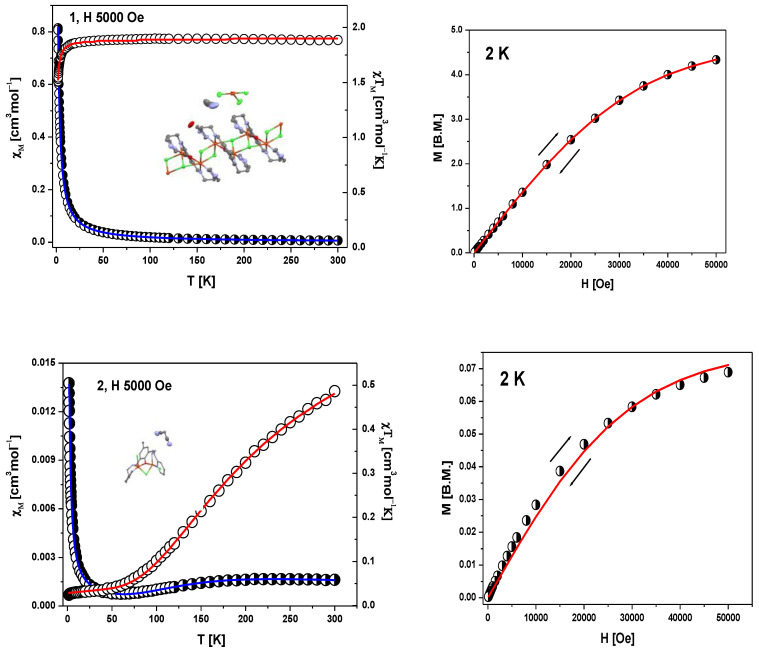
DC magnetic data for **1** and **2**; left panels: temperature dependence of χM and of the *χ*_M_*T* product. Right panels: field dependence of the magnetization per formula unit. The solid lines (on both graphs) are calculated using the HDVV spin Hamiltonian and PHI program [40].

The molar magnetic susceptibility for **1** and **2** complexes has been converted to the *χ*_M_*T* product (or effective magnetic moment) whose temperature dependence is displayed in Figure 6, left panels. The field dependence of the magnetization per formula unit *M*_1_ = *M*_mol_/*N*_A_*µ*_B_ at the constant temperature is shown in Figure 6, right panels.

The product function *χ*_M_*T* (and/or the effective magnetic moment) for **1** (Figure 6) remains almost constant upon cooling from room temperature down to *T* = 40 K; *χ*_M_*T* (300 K) = 1.87 cm^3^mol^−1^ K (*μ*_eff_ = 3.90 μ_B_) (a little bit higher than expected 1.85 cm^3^mol^−1^K (*μ*_eff_ = 3.87 μ_B_) for five Cu(II) ions with no exchange interactions with *S* = ^1^/_2_ and *g*_av_ = 2.00). Below 40 K, a slow decrease in the *χ*_M_*T* product is observed until 20 K, followed by a rapid decrease down to *χ*_M_*T* (1.8 K) = 1.51 cm^3^mol^−1^K.

The fitting of magnetic data of **1** was carried out using the PHI program, taking into account two different exchange pathways, through a double chloride bridge (*J*_1_) and a single oxide bridge (*J*_2_) and the TIP parameter based on spin Hamiltonian:

(1) $$H=\sum_{n=1}^{\frac{N}{2}}[-2J_{1}S_{2n-1}^{z}S_{2n}^{z}-2J_{2}S_{2n}^{z}S_{2n+1}^{z}-g\beta H(S_{2n-1}^{z}-S_{2n}^{z})]$$

where $S_{2n}^{z}$ denotes the *z*–component of the 2n−th spin in a chain.

The intermolecular interaction between copper(II) ions in the chain and unbound [CuCl_4_]^2−^ species are omitted due to rather long Cu⋅⋅⋅Cu separation (the shortest one is 9.32 Å). In the fitting procedure, we consider also two different values of the *g* parameter (*g*_1_ for square pyramidal environment of copper ions inside the chain and *g*_2_ for square planar geometry of [CuCl_4_]^2−^). The least squares fit of the experimental data by these expressions leads to the following results: *g*_1_ = 2.19, *g*_2_ = 2.10 and *J*_1_ = −0.31 cm^−1^, *J*_2_ = −0.38 cm^−1^ and TIP = 59 · 10^−6^. The discrepancy factor is 7.59 × 10^−7^. The variation of magnetization versus magnetic field at 2 K (Figure 6, right) clearly confirms S = ^5^/_2_ ground state in **1**.

In the case of **2**, molar magnetic susceptibility shows the broad maximum at 210 K. Starting with 210 K, a gradual decrease together with decreasing temperature was observed up to the minimum at 50 K (Figure 6). Below this temperature, χ_M_ increases again due to the presence of paramagnetic impurity, the exact nature of which is not known. The maximum at ~210 K is indicative of strong antiferromagnetic coupling between Cu(II) centers in the Cu_2_OCl moieties.

The corresponding plot of the *χ*_M_*T* value (or magnetic moment) [per two Cu(II) centres] vs. temperature shows a decrease from 0.49 cm^3^mol^−1^K (1.97 µ_B_) at 300 K (lower than expected for the spin–only value for two isolated coper(II) ions with *S* = ½ and g = 2.00) to 0.025 cm^3^mol^−1^ K (0.44 µ_B_) at 1.79 K. This magnetic feature for the both complexes indicates that an exchange interaction (much weaker for **1**) of the antiferromagnetic nature applies.

The exchange interaction between two Cu(II) ions (*S*_A_ = *S*_B_ = ½) in **2** was described by the model of binuclear units realized through a chloride and an oxide bridge together with various additional Cu⋅⋅⋅Cu intermolecular contacts transmitted through the hydrogen bond and π-π interactions which is described by the z*J*’ parameter (where z is the number of adjacent binuclear or paramagnetic species around a given binuclear unit). The calculations were based on the Heisenberg–Dirac–Van Vleck Hamiltonian in the zero field given by Equation (2).

(2) *Ĥ* = −*J*Ŝ_A_Ŝ_B_ − z*J*’ <*S*_z_> Ŝ_z_

describing the isotropic magnetic exchange interaction, antiferromagnetic for *J* < 0. The well–known PHI program [40] was used which allows for the simultaneous fitting of χ*T*(*T*) and *M*(*µ*_0_*H*) dependencies. The temperature independent paramagnetism (TIP) and the fraction of monomeric species (*x* = 1 for one uncoupled spin) were also included in the fitting procedure. The best agreement with the experimental magnetic data for **2** was obtained for *J* = −137 cm^−1^, *zJ*’ = −2.01 cm^−1^, *g* = 2.06, and *x* = 0.076 — we add this value according to paramagnetic impurities presence, TIP = 116 · 10^−6^ cm^3^mol^−1^, *R* = Σ[(*χT*)_exp_ − (*χT*)_calc_]^2^/Σ[(*χT*)_exp_]^2^ = 2.1 × 10^−7^ (red and blue lines in Figure 6). The calculated curve well matches the magnetic data.

The magnetization per formula unit *M*_1_ = *M*_mol_/(*N*_A_*μ*_B_) at *B* = 5 T and *T* = 2.0 K did not reach the saturation plateau. In such a case, the ground state equals 1 (S = 1) and the magnetization (per {Cu–Cu} unit) should saturate to the value of *M*_sat_ = 2.0 μ_B_. At higher magnetic fields, the obtained value is much smaller and proves antiferromagnetic exchange coupling between Cu(II) ions. Secondly, the magnitude of z*J*’ demonstrates that the inter-dimer interactions are not negligible.

One of aims of the present work was to synthesize and examine the role of organic ligands in the structure formation of Cu(II) Schiff bases complexes and its influence on the strength of magnetic interactions. Based on the crystal structure, the possible magnetic exchange pathways in **1** include interdimer coupling via double asymmetric chloro-bridges and intradimer interaction though the oxide bridge that can be either ferromagnetic or antiferromagnetic unless in **2** it can be realized by a double chloro-oxide bridge and also by interdimer hydrogen bonding contacts which are generally antiferromagnetic by nature. Magnetic data clearly show antiferromagnetic character in the both complexes (much stronger in **2**). The explanation of these properties corresponds with the theories presented so far. According to this, the simple magnetostructural correlations related the magnitude of exchange coupling (*J*) with the value of the bridging angle (Cu–Cl–Cu) (φ) [41,42,43] or (Cu–O–Cu) (φ_2_) [44,45] and Cu–Cl/Cu-O bridge distance (R) particularly expressed by the φ/R ratio, to more complicated factors such as geometry around the paramagnetic centers,[28,46,47] and angular distortions given by the dihedral angle (*τ*) between the equatorial planes. The double chloro-bridged dimers in the complex **1** belong to the SP-I (square pyramids sharing a basal edge with parallel basal planes) (Figure S14) class of compounds [35], which usually shows small ferromagnetic or antiferromagnetic coupling constants (Table S2) with magnetic interactions, according to Hückel calculations [48], occurring through a π* type interaction between the d_x_^2^_−y_^2^ Cu(II) orbital and the p orbital of the chlorine atoms. The extent of this magnetic coupling is determined by small structural deviations from the ideal rectangular Cu_2_Cl_2_ core. For small bridging angle values (lower than 90°) and short Cu–Cl bridge distances, the magnetic coupling is ferromagnetic [47]. When the φ and R values of the complex **1** [φ = 92.99° and R = 2.344; 2.786 Å] are used in this relationship, weak antiferromagnetic coupling is predicted, which is consistent with the experimental results and the known Cu(II) dimers involving a similar double chloro-bridge. The small magnitude of this interaction may be due to the asymmetry of the chloride bridge (two different R values, one much shorter—2.786 Å and the other, much longer—2.344 Å) resulting in the SP geometry distortion and an unusually long distance Cu ⋯ Cu (3.787 Å). However, the φ/R ratio analysis shows that for a φ/R value lower than 32.6 or higher than 34.8 [° Å^−1^] the exchange interaction is antiferromagnetic and for values falling in between these limits, the interactions are ferromagnetic. In the case of our dimer, the φ/R value is 33.38 or 39.67 which partially fits this trend. This may result from its geometry distortions. Finally, the weak antiferromagnetic character of **1** can also be attributed to the occurrence of interdimer exchange between dimeric units through the oxide ion. For dimers with planar or near planar Cu_2_O_2_ cores, the exchange constant (*J*) varies linearly with the Cu–O–Cu angle (φ). For φ > 97.5°, the interaction is predicted to be antiferromagnetic (*S* = 0 ground state), and for φ < 97.5°, the ground state equals 1 (*S* = 1) and interaction should be ferromagnetic. Angular distortions of LCu-O-CuL units may cause the magnetic interaction to become less antiferromagnetic at a given φ value as the dihedral angle (δ) between the two Cu_2_O_2_ planes is reduced from 180° [49]. Notably, the *J*_2_ value (corresponding to the exchange interaction through the oxide bridge) for the complex **1** is significantly less negative than the ones determined for related complexes due to their larger angular distortion and Cu···Cu separation. It is also worth noting that the both superexchange interaction in the chain structure of **1** are similar in terms of the sign as well as the value. Listing the selected structural and magnetic data for the complex **2** and a number of related complexes in Table S2 indicates that the observed strong antiferromagnetic coupling is a result of the Cu···Cu interaction transmitted through the oxide bridge. The *δ* value lower than 180° as well as a large Cu–O–Cu angle (106.26°) spend the limit typical of such an interaction. In the case of a possible Cu–Cl–Cu magnetic pathway, the structural parameter suggested ferromagnetic properties (bridging angle 81.31° lower than 90°, short Cu–Cl bridge distances), which is not compatible with experimental data.

The powder EPR spectra of the **1** and **2** complexes at the liquid nitrogen temperature show broad, almost isotropic lines centered at *g*_eff_ = 2.15 and 2.13, respectively. The peak-to-peak line widths are about 800 G for **1** and 210 G for **2** (Figure S15). Such broadening of EPR spectra and lack of immanent for Cu(II) complexes the *g* tensor anisotropy (due to Jahn–Teller distortion), are usually caused by spin-spin interactions between relatively close Cu(II) ions arranged in dimeric units in the both complexes. Because the spectra do not undergo the resolution of the signals resulting from the resonance transition between the spin states *M*_S_ = ±1.0 due to S = 1 of two Cu(II) magnetically coupled, neither the tensor *g* components nor the zero field splitting *D* parameter can be determined.

### 2.5. UV-Vis and Fluorescence Spectroscopy

**Figure 7:**
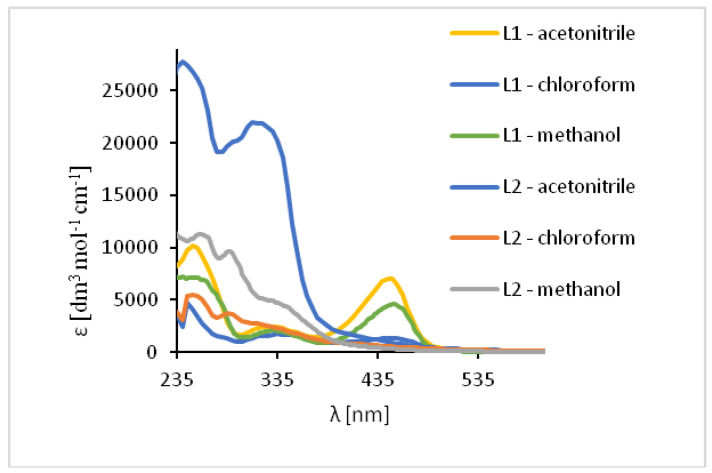
Solution absorption spectra of ligands in chloroform, acetonitrile and methanol, 1 × 10^−4^ mol/dm^3^, RT).

The absorption of UV-vis and fluorescence spectra of the ligands and their complexes were recorded at room temperature in various solvents of different polarity (MeCN, chloroform and methanol) (Figures 7 and 9–11, Table S3).

The UV-vis spectra of the **HL1** and **HL2** ligands exhibited bands between 245 and 254 nm, which are related to π → π* transitions of the benzene rings. (Figure 7, Table S3) Moreover, the bands in the range of 325–352 nm for **HL1** and 315–342 nm for **HL2** originated from HOMO → LUMO π → π* transitions of the azomethine group which was confirmed by DFT data (Table S4, Figures S16 and S17). Moreover, in the **HL2** the band registered at 290 nm is connected with the π → π* transitions of pyridine rings present in the ligand structure, what was previously observed [49]. Additionally, in the spectrum of **HL1** between 450 and 454 nm the band associated with charge transfer transition between the aromatic rings appeared. The emission of both ligands depends on the solvent polarity. The red shift of the fluorescence bands of the ligands **HL1**–**HL2** ligands along with the increase in solvent polarity was noted. This can be caused by the distortion of the molecule geometry in the excited state, what implies decrease of the resonance energy and bathochromic shifts (Figure 7, Table S3).

**Figure 8:**
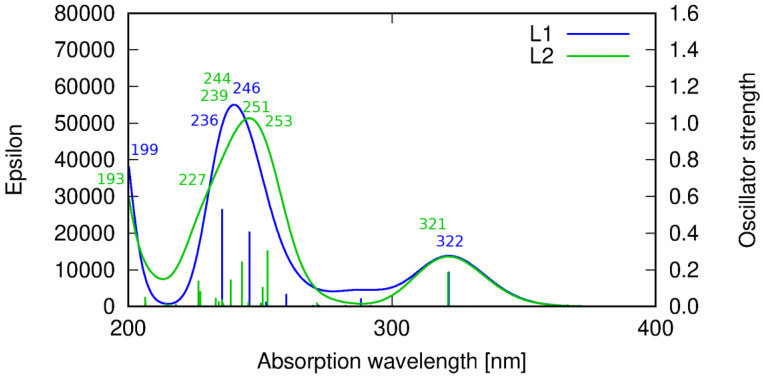
Theoretical prediction of the absorption spectrum of ligands **HL1** (blue line) and **HL2** (green line) in acetonitrile calculated with the PBE0 functional. Sticks correspond to the precise theoretical transition wavelength and oscillator strength, while the convoluted spectrum is obtained as their Gaussian fit.

The interpretation of the experimental spectra of the both ligands was additionally confirmed by the DFT calculations (Table S4, Figures S16–S19). Theoretical results indicate that the absorption spectra for **HL1** and **HL2** are comparable to each other due to a similar structure of the molecular skeleton of the both molecules and the nature of the consequent excitations. **HL1** exhibits the longest wavelength signal at 322 nm. This transition corresponds to the HOMO → LUMO excitation of the π → π* character. In the shorter wavelength range, additional multiple transitions are present, contributing to the more intensive band than that at about 320 nm (Figure 8).

For **HL1**, the signal at 246 nm corresponds to the transition of the dominating HOMO-4 → LUMO being π and π* orbitals, respectively, at the phenyl moiety. The **HL1** band at 236 nm arises from the HOMO-5 → LUMO transition of the π → π* character. All of these transitions involve the orbitals localized on the central phenyl ring and side chains fragments reaching up to the imine nitrogen (Table S4). On the other hand, the shortest wavelength signal for **HL1** included in the present considerations corresponds to the HOMO → LUMO+2 transition that involves the transition of the charge density between the central phenyl moiety and one of the side imidazole rings. The other imidazole ring contributes to the above transition in a slightly decreased fraction due to the lack of the whole molecule symmetry (the hydroxyl group pointing in the direction of one of the side chains).

Similar spectrum features are observed for **HL2**. The low intensity signal at 321 nm arises from the HOMO → LUMO transition of the π → π* character, as for **HL1**. Next, towards the shortened wavelength, a more intensive band which consists of several signals appears. At 253 nm the HOMO-1 → LUMO transition of the π → π* type can be noticed, followed by the HOMO-6 → LUMO+3 transition for λ = 251 nm, involving π and π* orbitals on the pyridine rings. At 244 nm, another π → π* transition localized at the central phenyl ring moiety is observed. The HOMO-3 → LUMO transition appearing at 239 nm is characterized by the charge transfer between the pyridine and the phenyl rings. A 227 nm transition corresponds to the π → π* excitation at the heterocyclic ring, while the shortest wavelength (193 nm) band involves the π → π* orbitals at the phenyl ring.

**Figure 9:**
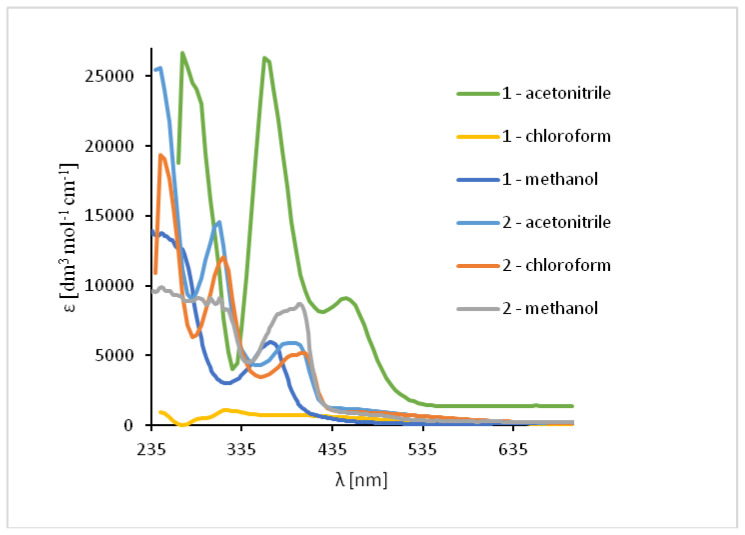
Solution absorption spectra of complexes in chloroform, acetonitrile and methanol, 1 × 10^−4^ mol/dm^3^, RT).

In the UV-Vis spectra of Cu(II) compounds, the most characteristic bands for the copper(II) Schiff base complexes, assigned to π → π* transitions in the -C=N- group were registered in the region of 320–372 nm for **1** and 312–315 nm for **2** in dependence on the solvent. (Figure 9, Table S3) They were shifted towards higher wavelengths in comparison to the free ligand (∆ = 38–43 nm for **1**) and towards lower wavelengths (∆ = 5–30 nm for **2**). These shifts confirm the coordination of the ligand with the copper(II) ion. In the complex **1** the dependence of the shift of these bands on the solvent polarity was not observed but in **2**, the value of a shift increased together with a decrease in the solvent polarity. Additionally, in the absorption spectra the presence of low intensity absorption bands from *d-d* transitions (*d*_xz_, *d*_yz_ → *d*_x2−y2_) [50,51] between 575–666 nm (**1**) and in the range of 500–626 nm (**2**) was noted. This results from the square pyramidal environment of the copper atoms shifted towards the apical chloride anion in **1** and significantly deformed square pyramidal geometry with the shared edge and the non-bridging Cl1 and Cl2 in the apical positions of the copper ions in **2**. The same was observed previously for similar copper(II) compounds [27].

**Figure 10:**
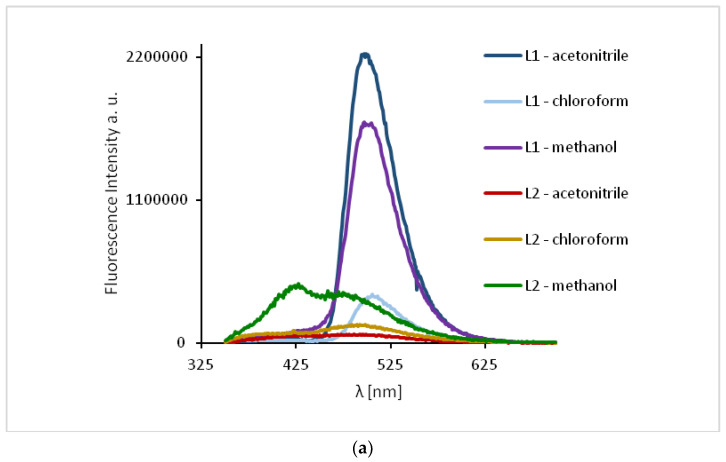

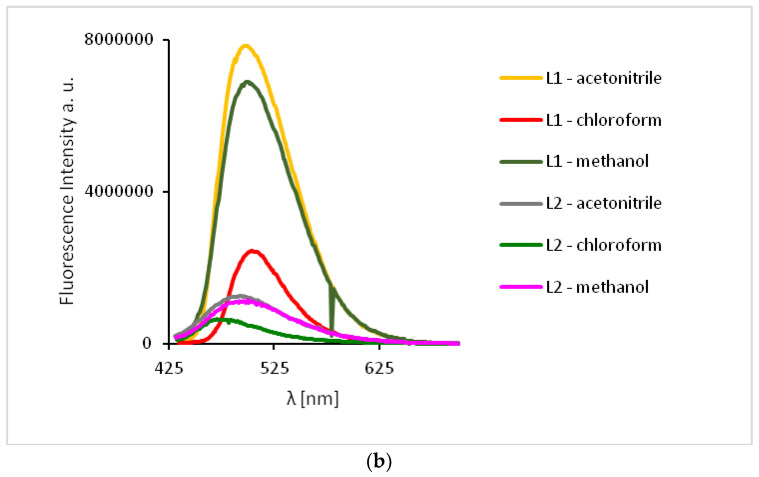
Solution emission spectra of ligands **HL1** and **HL2**. (**a**) *λ*_ex_ = 296 nm, (**b**) *λ*_ex_ = 422 nm, (MeCN, chloroform and methanol 1 × 10^−4^ mol/dm^3^, RT).

The study of the ligands fluorescence properties showed emission between 497–506 nm (**HL1**) and 470–492 nm (**HL2**) (*λ*_ex_ = 296 nm), while excitation at 422 nm led to a stronger emission intensity in the range of 498–503 nm for **HL1** and 471–498 nm for **HL2** (Figure 10, Supplementary Table S3). Slight hipsochromic shifts of the emission bands together with a decrease in the solvent polarity were registered. It can be related to the geometry distortion in the excited state, which implies a decrease in the resonance energy.

**Figure 11:**
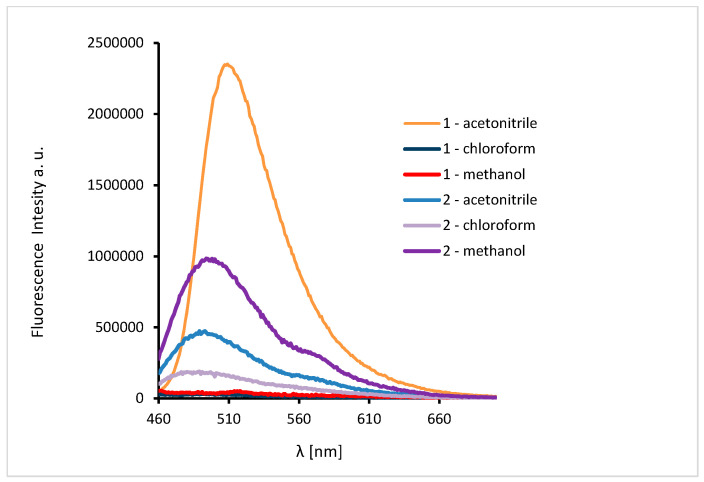
Solution emission spectra of complexes. (*λ*_ex_ = 450 nm, MeCN, chloroform and methanol 1 × 10^−4^ mol/dm^3^, RT).

The excitation of **1** and **2** in all the solvents at 450 nm resulted in blue emission between 497–509 nm for **1** and 489–500 nm for **2** (Figure 11). For the both complexes, slight hipsochromic shifts of emission bands were observed when shifts of the ligands in all the solvents were compared. This can be caused by lowering the ligand lability and changes in the molecular geometry of the excited state, which implies a decrease in the resonance energy. Spectra of **1** and **2** exhibit an increase in the fluorescence intensity in polar solvents and bathochromic shifts with increasing solvent polarity. The fluorescence intensity of **HL2** and **2** was significantly lower in comparison with that of the solutions of **HL1** and **1**, respectively (Figure 10 and Figure 11). This can result from the presence of the chloride bridge between the copper centers, which then causes lowering of the ligand lability, a different pathway of non-radiative transition, and finally, lowering of the fluorescence intensity.

### 2.6. Thin Films of Copper(II) Complexes

The morphology and the surface roughness of the thin films were investigated by SEM and AFM techniques. In order to study the chemical composition of the films EDS analyses were conducted for all samples. The optimum parameters of layers (roughness, thickness, and homogeneity) were obtained at multistage spin coating process, spin speed 3000 rpm, time of coating 5 s.

**Figure 12:**
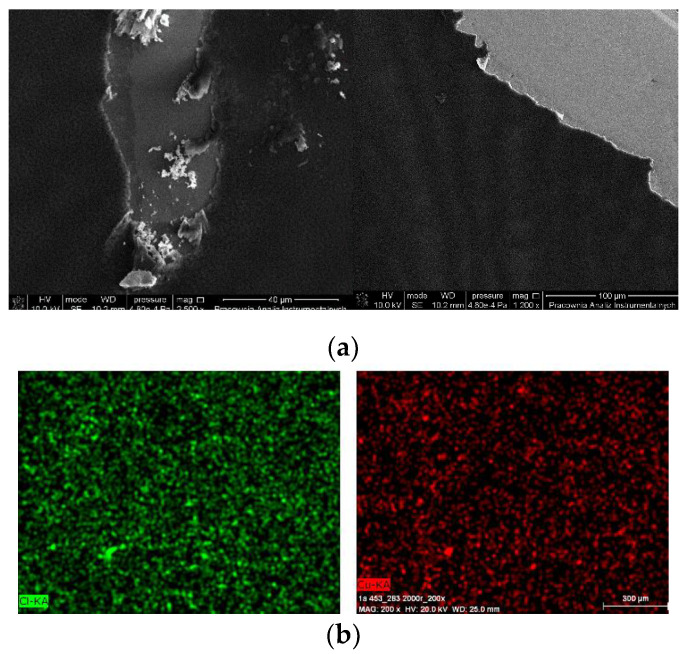
SEM of (**a**) {[Cu_2_(L1)Cl_2_]_2_[CuCl_4_]}·2MeCN·2H_2_O **1** /PMMA/Si PMMA 3000 rpm 5 s, 3000 rpm PMMA+ complex **1** × 2 {[Cu_2_(L1)Cl_2_]_2_[CuCl_4_]}·2MeCN·2H_2_O **1** 3000 rpm ×4, 5 s, (**b**) EDS mapping of [Cu_2_(L2)Cl_3_] 0.5 MeCN·**2**/PMMA/Si, scan size 1 μm, 3000 rpm PMMA ×1, complex **2** 3000 rpm ×2, complex + PMMA 3000 rpm ×1, complex 3000 rpm ×1, 5 s.

The thin, homogeneous films of complexes were obtained at silicon surfaces first immobilized with PPMA. The visible scratches on the surface of the film in the SEM images show the edge of the thin complex layer, which clearly indicate the presence of the copper(II) complex layers what was additionally confirmed with the EDS results (Cu 6.80 mass%, Cl 5.69 mass%). Moreover, the mapping EDS confirmed the presence of copper(II) complex over the entire layer (Figure 12b).

**Figure 13:**
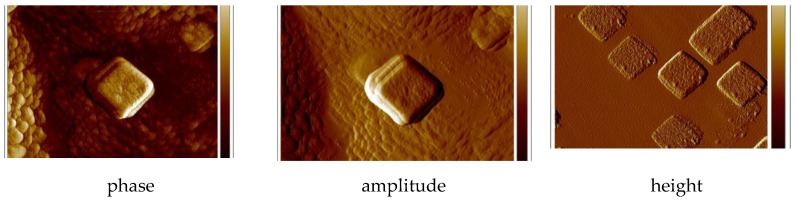
AFM of {[Cu_2_(L1)Cl_2_]_2_[CuCl_4_]}·2MeCN·2H_2_O (**1**), PMMA/Si; PMMA ×1, PMMA + {[Cu_2_(L1)Cl_2_]_2_[CuCl_4_]}·2MeCN·2H_2_O (**1**) × 1, {[Cu_2_(L1)Cl_2_]_2_[CuCl_4_]}·2MeCN·2H_2_O (**1**) ×5, 2000 rpm 5 s scan size 1 μm, height (thickness) 25 nm, R_a_ = 8.85–19.9 nm, R_q_ = 11.1–25.9 nm.

The two-dimensional (2D) and three dimensional (3D) AFM images scanned over a surface area of 1 × 1 µm^2^ are shown in Figure 13 and Figures S20 and S21. The root-mean square (RMS) parameters were calculated from AFM images. The AFM image of the films indicate thin, amorphous layers of both copper(II) complexes deposited on the silicon surfaces with roughness parameters ranged R_a_ = 4.40–6.15 nm and R_q_ = 6.43–7.94 nm for **1** and **2**, respectively. (Figure 13, Figures S20 and S21) The values of roughness parameters indicate that the obtained films are smooth. The phase images of the complexes, PMMA/Si show the presence one layer of compound deposited on entire Si surface and dense morphology of the layer. Additionally, the layer of complex **2** consists of quasi regular square structures with dimensions 0.28 × 0.3 μm.

Height (thickness) of the materials equaled 25 nm. Additionally, the influence of the covering condition was noted. The PMMA usage significantly improved the quality of the new copper(II) films, what was observed also previously by us [5,32].

**Figure 14:**
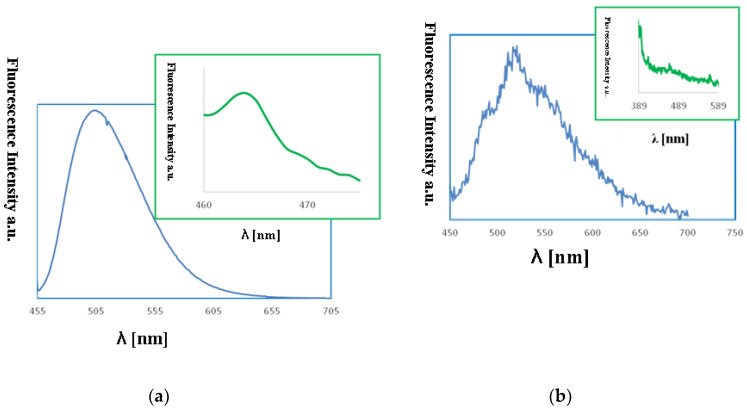
Fluorescence spectra of (**a**) {[Cu_2_(L1)Cl_2_]_2_[CuCl_4_]}·2MeCN·2H_2_O (**1**), MeCN, *λ*_em_ = 509 nm blue lines (**b**) {[Cu_2_(L1)Cl_2_]_2_[CuCl_4_]}·2MeCN·2H_2_O, PMMA/Si λ_ex_ = 450 nm, λ_em_ = 464 nm, green lines (**a**): [Cu_2_(L2)Cl_3_]⋅0.5MeCN (**2**), MeCN, *λ*_ex_ = 440 nm, *λ*_em_ = 520 nm, (**b**) [Cu_2_(L2)Cl_3_]⋅0.5MeCN, PMMA/Si, λ_ex_ = 380 nm, λ_em_ = 460 nm.

The fluorescence properties of the copper(II) complexes films were also studied. The emission of the copper(II) films was observed between 460–464 nm (*λ*_ex_ from 380 to 450 nm) and was connected with the LMCT transitions (Figure 14). The bathochromic shift of the florescence bands of the films in comparison to the solution was registered. An influence of molecular packing in the solid phase on the optical properties can therefore be concluded. This can arise from a different pathway of the non-radiative transitions. It can be related to the reduction of the ligand conformational flexibility in the complex. This reduction results from the restraints imposed by the substrate surface. The copper(II) layers **1**, PMMA/Si exhibited high fluorescence intensity. The highest intensity for the smooth, thin layer with the equally distributed complex **1** on Si surface obtained at 3000 rpm min^−1^ was noted (Figure 14). In contrast, the **2**, PMMA/Si films showed very weak fluorescence intensity. These results are compatible with the fluorescence emission observed for the **1** and **2** compounds in solutions, where the same trend in fluorescence intensity was noted. 

A preparation of thin films by spin coating, provides advantages in layer homogeneity and in fluorescence of the obtained samples. The new high ordered copper(II) films of **1** with fluorescent properties are promising candidates for solar cells applications.

## 3. Materials and Methods

2-hydroxy-5-methylisophthalaldehyde (97%), histamine (98%), 2-(2-pirydyl)ethylamine (97%) were purchased from Aldrich and used without further purification. Copper(II) chloride dihydrate, (analytical grade) was supplied by POCH (Gliwice Poland).

### 3.1. Methods and Instrumentation

^1^H, ^13^C, ^1^H ^13^C hmqc and hmbc NMR spectra of the ligands were collected with Bruker Avance III 400 MHz or Bruker Avance 700 MHz spectrometers in CDCl_3_ or DMSO-_d6_. UV-vis absorption spectra were recorded on a Hitachi spectrophotometer in CH_3_CN, MeOH or chloroform (1 × 10^−4^ M) solutions. The fluorescence spectra were recorded on a spectrofluorimeter Gildenpλotonics 700 in the range 900–200 nm (MeCN, chloroform and methanol solutions of compounds the same as in the case of UV-vis studies or silicon slides). The elemental analysis was carried out using Vario EL III Elemental analyzer. The thermal analysis (TG, DTG, DTA) was performed on an SDT 2960 TA analyzer under air, heating rate 10 °C min^−1^, and heating range up to 1000 °C. The IR spectra were performed on the FT-IR Vertex 70V equipped with Hyperion 1000/2000 Bruker Optik using the ATR technique in the range 70–4000 cm^−1^.

The complexes layers were deposited on Si(111) wafers (10 nm × 10 mm) ~500 nm thick using the spin coating technique. Precursors were dissolved in acetonitrile and deposited on Si using a spin coater (Laurell 650 SZ). PMMA (poly(methyl-2-methylpropenoate) was used to improve adhesion to the silicon surface. The spin speed was varied from 2000 rpm to 3000 rpm, the coating time was 5 s. The morphology and composition of the obtained films were analyzed with a scanning electron microscope, (SEM) LEO Electron Microscopy Ltd., England, model 21430 VP equipped with secondary electrons (SE) detectors and an energy dispersive X-ray spectrometer (EDX) Quantax with a XFlash 4010 detector (Bruker AXS microanalysis GmbH). The layers morphology was also studied using SEM/FIB (scanning electron microscope/focused ion beam) Quanta 3D FEG equipped with the gold and palladium splutter SC7620. The atomic force microscopy (AFM) images were performed in the tapping mode with a MultiMode Nano Scope IIIa (Veeco Digital Instrument) microscope.

Magnetic measurements: Variable–temperature (2–300 K) direct current (DC) magnetic susceptibility measurements under applied field of 0.1 (*T* < 20 K) and 1.0 kG (*T* ≥ 20 K) and variable–field (0–5 T) magnetization measurements at low temperatures in the range 2 K were carried out with Quantum Design SQUID magnetometer. Raw magnetic susceptibility data were corrected for the underlying diamagnetism [52] and the sample holder. Magnetic measurements were carried out by crushing the crystals and restraining the sample in order to prevent any displacement due to its magnetic anisotropy. The EPR spectra were measured using a Bruker Elexys E 500 Spectrometer equipped with NMR teslameter and frequency counter at X−band and at 77 K.

### 3.2. Crystal Structure Determination

For a single crystal, the diffraction data of **1** were collected at room temperature using Oxford Sapphire CCD diffractometer, MoKα radiation *λ* = 0.71073 Å, whereas for **2** at 100 K on MX14.2 beamline (Helmholtz Zentrum Berlin, Bessy II) operating at *λ* = 0.82657 Å. In CrysAlis [53] we found that the selected crystal was twinned and the data were processed with two domains for **1**. However, the number of the unindexed spots points that more domains could be proposed, but any attempts to find a third reasonable domain failed. We applied the analytical absorption correction for a two domain model. For **2**, the data were initially processed using *xdsapp* [54,55] software, and subsequently, the numerical absorption was applied in CrysAlis Pro.[54] The both structures were solved by the direct methods and refined with the full-matrix least-squares procedure on F^2^ with HKLF 5 and HKLF 4 flags for **1** and **2**, respectively (SHELX-97 [56]). All the heavy atoms were refined with the anisotropic displacement parameters. The hydrogen atoms positions were assigned at the calculated positions with thermal displacement parameters fixed to a value 20% or 50% higher than those of the corresponding carbon atoms. In **1**, for the C32 atom restraints (ISOR) were applied. In the final model, there is lack of hydrogen atoms from the O34 crystallization water molecule. Due to complicated twinning, electron density peaks are still observed. They reach 2.3 electrons and cannot be ascribed to any new atom as they are very close to the position of the atom already existing in the model. For **2**, the acetone molecule with partial occupancy (0.5) and the methyl group located in the proximity of a twofold axis were found. All the figures were prepared in DIAMOND [57] and ORTEP-3 [58]. The results of the data collection and refinement have been summarized in Table 1 and Table 2.

CCDC 1873388 and 1997646 contain the supplementary crystallographic data for **1** and **2**, respectively. These data can be obtained free of charge via http://www.ccdc.cam.ac.uk/conts/retrieving.html, or from the Cambridge Crystallographic Data Centre, 12 Union Road, Cambridge CB2 1EZ, UK; fax: (+44) 1223-336-033; or e-mail: deposit@ccdc.cam.ac.uk. Supplementary data associated with this article can be found, in the online version.

### 3.3. Computational Details

Full optimization of the ligand geometry was carried out with the B3LYP/def2-TZVP approach both in gas phase and in PCM solvent (acetonitrile and dimethylsulfoxide). The def2-TZVP basis set was applied throughout all ligand calculations. The absorption spectrum for ligands was estimated within the B3LYP, PBE0, and CAM-B3LYP functionals in vertical approach in the linear response formalism for both solvents. In the main text of the manuscript only PBE0 results in acetonitrile are given due to their best correspondence to the experimental data. All the remaining results are available in Supplementary Information. NMR spectrum for ligands was calculated with the B3LYP functional. All ligand calculations were performed with Gaussian16 program [59].

## 4. Experimental

### 4.1. Synthesis of Ligands

**HL1 (Figure 15)**

To the 2-hydroxy-5-methyl-1,3-benzenedicarboxaldehyde (0.2396 g; 1.46 mmol) in warm ethanol histamine dihydrochloride (0.5374 g; 2.92 mmol) in sodium hydroxide solution (0.1200 g in 10 mL water) was added. The reaction mixture was refluxed for 4 h and the solvent was removed on a rotary evaporator. The product was dried under air, received orange oil (m = 0.3902 g, 1.11 mmol Yield 96%). C_19_H_22_N_6_O (calc./found%): C 65.12/65.41, N 23.97/24.11, H 6.32/6.29.

^1^H [ppm]: 2.21 (s, 3H) H1, CH_3_, 3.16 (m, 2H) (H8), CH_2_, 4.10 (m, 2H) (H7), CH_2_, 5.75 (s, 1H) N-H, 8.48 (s, 1H) -N=CH-, 10.32 (s, 1H) -OH, 7.29 (d, 1H) Ar-H (H10), 7.48 (d, 1H) Ar-H (H3), 7.69 (d, 1H) Ar-H (H12). ^13^C [ppm]: 20.07 (C1), 37.83 (C8), 51.43 (C7), 161.03 (C6), 122.30 (C12), 119.59 (C10), 117.07 (C4), 139.76 (C2), 132.50 (C3), 160.54 (C5), 134.19 (C9). Selected FT-IR (data reflectance, oil) (cm^−1^) 3372 ν_OH_, 3096, 3000, 2842, ν_C-HAr_, 1663 ν_C=N_, 1635, 1524ν_C=CHA_, 1461, 1372 ν_C=CAr_, 1339 ν_C-NHA_, 1280 ν_Ph-O_, 1191. UV-vis (MeCN, 1 × 10^−4^ mol/dm^3^): *λ*/nm 250 (*ε*/dm^3^ mol^−1^ cm^−1^ 10180), 325 (2460), 450 (6970), (chloroform, 1 × 10^−4^ mol/dm^3^): *λ*/nm 245 (*ε*/dm^3^ mol^−1^ cm^−1^ 4720), 352 (1569), 455 (1290), (MeOH, 1 × 10^−4^ mol/dm^3^): *λ*/nm 254 (*ε*/dm^3^ mol^−1^ cm^−1^ 7116), 334 (2056), 454 (4522).[60]

**HL2 (Figure 16)**

2-hydroxy-5-methyl-1,3-benzenedicarboxaldehyde (0.8208 g; 5 mmol) and 2-(2-pirydyl)ethylamine (1.26 mL; 10 mmol) were stirred in methanol for 3 h at RT (orange solution), then to the mixture sodium tetrafluoroborate (0.7600 g) and sodium hydroxide (0.3700 g) in 5 mL water were added. After 6 h stirring the reaction mixture (yellow solution) was reduced in volume on a rotary evaporator and extracted with chloroform (3 × 25 mL). The combined extracts were dried on anhydrous magnesium sulfate. The solution was filtered and the solvent was removed on a rotary evaporator. The product was a yellow oil (m = 1.7506 g, 4.7 mmol, Yield 94%). C_23_H_24_N_4_O (calc./found%): C 74.16/74.19, N 15.04/14.96, H 6.49/6.42.

^1^H [ppm]: 2.21 (s, 3H) CH_3_, 3.16 (m, 2H) CH_2_, 4.10 (m, 2H) CH_2_, 8.48 (s, 1H) -N=CH-, 7.29 Ar-H, 7.48 (m, 1H) Ar-H, 7.69 (m, 2H) Ar-H, 8.95 (m, 1H) Ar-H, 10.32 (OH), 5.75 (s, 1H) NH. ^13^C [ppm]: 21.42 (C1), 49.36 (C8), 51.70 (C7), 167.44 (C6), 122.30 (C12), 119.59 (C10), 125.27 (C4), 128.45 (C2), 129.59 (C3), 137.39 (C11), 150.27 (C13), 165.56 (C5), 161.03 (C9).[36,60] Selected FT-IR (data reflectance, oil) (cm^−1^) 3359, 3283 ν_OH_, 3050, 3009, 2919, 2849 ν_C-HAr_, 1672 ν_C=N_, 1592, 1569ν_C=CHA_, 1476, 1436 ν_C=CAr_, 1361, 1302ν_C-NHA_, 1252 ν_Ph-O_, 1153. UV-vis (MeCN, 1 × 10^−4^ mol/dm^3^): *λ*/nm 254 (*ε*/dm^3^ mol^−1^ cm^−1^ 27400), 290 (20110), 315 (21830), (chloroform, 1 × 10^−4^ mol/dm^3^): *λ*/nm 255 (*ε*/dm^3^ mol^−1^ cm^−1^ 5400), 290 (3570), 335 (2310), (MeOH, 1 × 10^−4^ mol/dm^3^): *λ*/nm 262 (*ε*/dm^3^ mol^−1^ cm^−1^ 11134), 290 (9448), 342 (4426).

### 4.2. Synthesis of Complexes

**{[Cu_2_(L1)_2_Cl_2_]_2_[CuCl_4_]}·2MeCN·2H_2_O (** **1)**

Copper(II) chloride dihydrate (0.8520 g; 5 mmol) in EtOH (20 mL) was added to an ethanolic solution (10 mL) of the ligand **HL1** (0.8760 g; 2.5 mmol). The mixture was stirred for 4 h at RT. The resulting dark green filtrate was slowly evaporated to dryness. Dark black crystals suitable for X-ray data collection from CH_3_Cl and MeCN after recrystallization were isolated (m= 1.3695 g, 0.96 mmol, Yield: 38.5%). C_42_H_52_Cl_8_Cu_5_N_14_O_4_ (calc./found%): C 35.57/35.21, N 13.83/11.90, H 3.70/4.09.

Selected FT-IR (data reflectance, solid crystal) (cm^−1^): 3527 ν_OH_, 3189, 3142, 3041, 2970 ν_C-HAr_, 1641 ν_C=N_, 1568, 1500 ν_C=CHA_, 1451, 1405ν_C=CAr_, 1346, 1332ν_C-NHA_, 1216 ν_Ph-O_, 1168, 1078, 1030. UV-vis (MeCN, 1 × 10^−4^ mol/dm^3^): *λ*/nm 270 (*ε*/dm^3^ mol^−1^ cm^−1^ 12,300), 365 (26,050), 455 (8990), 650 (1410), (chloroform, 1 × 10^−4^ mol/dm^3^): *λ*/nm 320 (*ε*/dm^3^ mol^−1^ cm^−1^ 1060), 395 (730), 575 (240), (MeOH, 1 × 10^−4^ mol/dm^3^): *λ*/nm 272 (*ε*/dm^3^ mol^−1^ cm^−1^ 12,300), 372 (5800), 666 (165).

**[Cu_2_(L2)Cl_3_]0.5MeCN (2)**

Copper(II) chloride dihydrate (0.2249 g; 1.32 mmol) in EtOH (10 mL) was added to an ethanolic solution (10 mL) of the ligand **HL2** (0.2458 g; 0.66 mmol). The reaction mixture was stirred for 4 h at RT. The obtained brown precipitate was filtered and dried under air. The crystals suitable for X-ray data collection from CH_3_Cl and MeCN after recrystallization were isolated (m= 0.1764 g, 0.27 mmol, Yield: 42.6%). C_24_H_24.5_Cl_3_Cu_2_N_4.5_O (calc./found%): C 46.09/46.25, N 10.08/9.87, H 3.95/4.05. Selected FT-IR (data reflectance, solid crystal) (cm^−1^) 3126, 3028, 2943, 2902 ν_C-HAr_, 1609 ν_C=N_, 1570, ν_C=Cpy_, 1478, 1445, 1422 ν_C=CAr_, 1332, 1316 ν_C-Npy_, 1251 ν_Ph-O_, 1161, 1112. UV-vis (MeCN, 1 × 10^−4^ mol/dm^3^): *λ*/nm 245 (*ε*/dm^3^ mol^−1^ cm^−1^ 25,600), 310 (14,560), 395 (5880), 500 (910), (chloroform, 1 × 10^−4^ mol/dm^3^): *λ*/nm 250 (*ε*/dm^3^ mol^−1^ cm^−1^ 19,070), 315 (12,080), 400 (5150), 575 (240), 665 (120) (MeOH, 1 × 10^−4^ mol/dm^3^): *λ*/nm 248 (*ε*/dm^3^ mol^−1^ cm^−1^ 9836), 312 (9007), 402 (8511), 546(3 42), 626 (271).

## 5. Conclusions

Two copper(II) complexes, **1** and **2**, with Schiff bases derived from 2-hydroxy-5-methylisophthalaldehyde and histamine **HL1** or 2-(2-aminoethyl)pyridine **HL2** 1-[(2-pyridin-2-yl-ethylimino)-methyl]-naphthalen-2-ol, respectively, were obtained. In spite of a similar NNO tridentate character of the Schiff bases and owing the bridging oxygen of the ligand and chloride ions, the dimerization of Cu(II) ions leads to creation different molecular and crystal structures. The complex **1**, consists of [Cu2(L1)Cl2]^+^ units in which Cu(II) ions are bridged by the ligand **HL1** oxygen and each of these Cu(II) ions are connected with Cu(II) ions of the next dimeric unit via two bridging Cl^−^ ions to finally form a chain structure. In the dinuclear complex **2**, each Cu(II) is asymmetrically bridged by the ligand **HL2** oxygen and a chloride anion, whereas the remaining chloride anions are bound to Cu(II) cations at the axial positions. The penta-coordinate arrangement of the ligands around Cu(II) centers resulted in a geometry close to square - planar in **1**, whereas in **2**, it was distorted to a much greater degree (with τ_5_ = 0.413 and 0.391 for each Cu(II) ion. The magnetic studies have shown that antiferromagnetic interactions are strong (*J* = −137 cm^−1^) in the complex **2** and weak in **1** (*J*_1_ = −0.31 cm^−1^, *J*_2_ = −0.38 cm^−1^) which could be explained by magneto-structural correlations. The EPR spectra of the complexes **1** and **2** show a broad, almost isotropic lines centered at *g*_eff_ = 2.15 and 2.13 which are unusual for Jahn-Teller distorted Cu(II) complexes geometries; the spectra are characteristic of the unresolved EPR signals for the systems with *S* > 1/2 due to spin-spin interactions between metal ions. The compliance between experimental and theoretical results has validated the developed calculation method which will be used to design new binuclear copper(II) complexes. The highly ordered layers were obtained and exhibited fluorescence in the range of 460–464 nm. The bathochromic shift of the fluorescence bands of films in comparison to that of the solution was noted. This can result from molecular packing in the solid state being different to that in the solution. The fluorescence emission of the layers makes these films potentially suitable for application in light emitting devices.

## Figures and Tables

**Figure 15:**
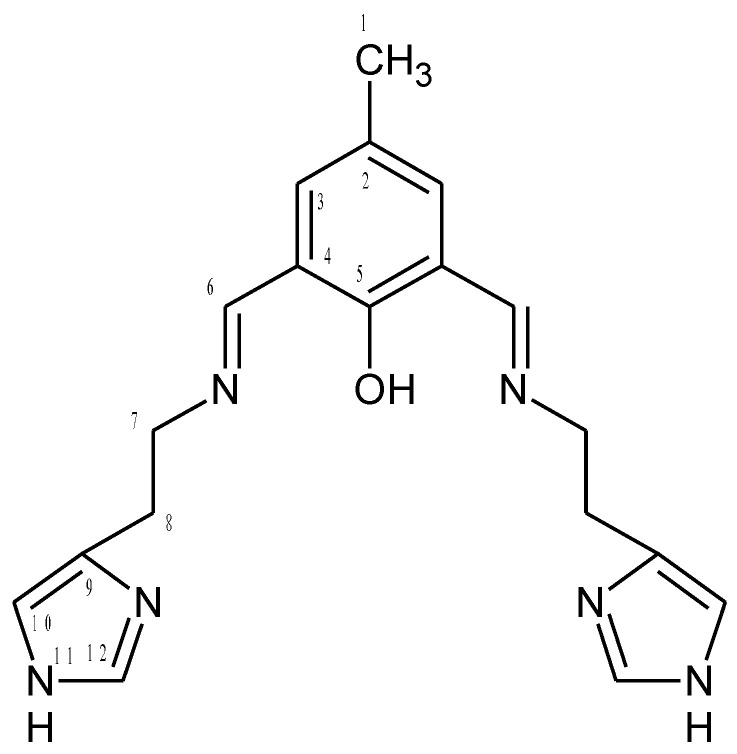
Structural formula and atomic numbering scheme for **HL1**.

**Figure 16:**
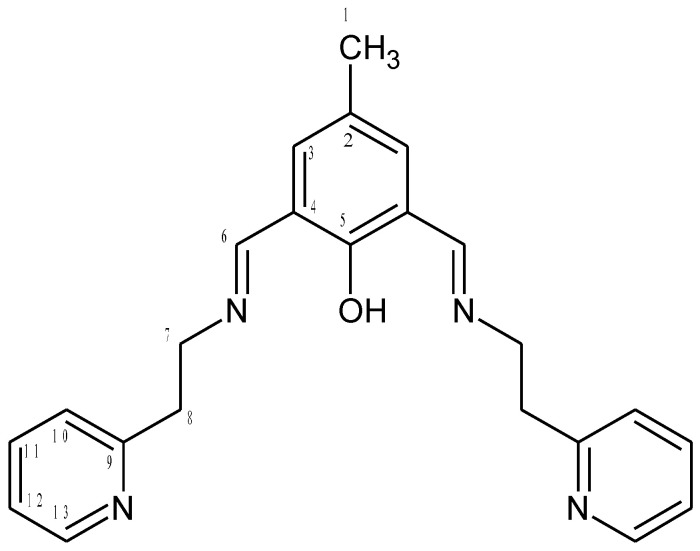
Structural formula and atomic numbering scheme for **HL2.**

**Table 1:** Selected bond lengths [Å] and valence angles [°] for **1** and **2**.

1				2			
Cu1-O1	1.956(5)	Cu2-O1	1.959(5)	Cu1-O1	1.954(2)	Cu2-O1	1.982(2)
Cu1-N30	1.962(6)	Cu2-N10	1.959(7)	Cu1-N30	1.997(3)	Cu2-N10	2.004(3)
Cu1-N23	2.025(7)	Cu2-N3	2.006(6)	Cu1-N23	2.055(3)	Cu2-N3	2.024(3)
Cu1-Cl1	2.350(2)	Cu2-Cl2	2.344(2)	Cu1-Cl3	2.3926(8)	Cu2-Cl2	2.3906(9)
Cu1-Cl1 ^i^	2.849(2)	Cu2-Cl2 ^ii^	2.786(2)	Cu1-Cl1	2.4254(9)	Cu2-Cl3	2.4279(8)
Cu3-Cl4	2.274(3)	Cu3-Cl3	2.275(3)	Cu1-Cl3-Cu2	79.87(3)		
Cu3-Cl4 ^i^	2.274(3)	Cu3-Cl3 ^i^	2.275(3)	O1-Cu1-N30	167.08(11)	O1-Cu2-N10	163.35(10)
O1-Cu1-N30	169.7(3)	O1-Cu2-N10	168.8(3)	O1-Cu1-N23	92.61(11)	O1-Cu2-N3	85.37(11)
O1-Cu1-N23	88.7(3)	O1-Cu2-N3	87.5(3)	N30-Cu1-N23	94.08(12)	N10-Cu2-N3	95.82(12)
N30-Cu1-N23	93.0(3)	N10-Cu2-N3	92.9(3)	O1-Cu1-Cl3	80.32(7)	O1-Cu2-Cl2	96.56(8)
O1-Cu1-Cl1	86.27 (18)	O1-Cu2-Cl2	86.30(17)	N30-Cu1-Cl3	87.64(8)	N10-Cu2-Cl2	98.64(8)
N30-Cu1-Cl1	93.5(2)	N10-Cu2-Cl2	94.0(2)	N23-Cu1-Cl3	142.27(9)	N3-Cu2-Cl2	109.63(9)
N23-Cu1-Cl1	169.9(2)	N314-Cu2-Cl2	172.3(2)	O1-Cu1-Cl1	92.43(8)	O1-Cu2-Cl3	78.91(7)
O1-Cu1-Cl1 ^i^	93.01(16)	O1-Cu2-Cl2 ^ii^	94.69(16)	N30-Cu1-Cl1	96.15(9)	N10-Cu2-Cl3	89.80(8)
N30-Cu1-Cl1 ^i^	97.2(2)	N10-Cu2-Cl2 ^ii^	96.5(2)	N23-Cu1-Cl1	107.05(9)	N3-Cu2-Cl3	139.87(9)
N23-Cu1-Cl1 ^i^	84.5(2)	N3-Cu2-Cl2 ^ii^	88.8(2)	Cl3-Cu1-Cl1	110.23(3)	Cl2-Cu2-Cl3	108.69(3)
Cl1-Cu1-Cl1 ^i^	87.01(7)	Cl2-Cu2-Cl2 ^ii^	87.19(7)				
Cl4-Cu3-Cl4 ^i^	180.0	Cl4-Cu3-Cl3 ^i^	89.64(10)				
Cl4-Cu3-Cl3	90.36(10)	Cl4 ^i^-Cu3-Cl3 ^i^	90.36(10)				
Cl4 ^i^-Cu3-Cl3	89.64(10)	Cl3-Cu3-Cl3 ^i^	180.0				

(**1**) ^i^ −*x*, −*y* + 1, −*z* + 2; ^ii^ −*x* + 1, −*y* + 1, −*z* + 2.

**Table 2:** Crystal data and structure refinement for **1** and **2**.

Identification Code	1 CCDC 1873388	2 CCDC1997646
Empirical formula	C_42_H_52_Cl_8_Cu_5_N_14_O_4_	C_24_H_24.50_Cl_3_Cu_2_N_4.50_O
Formula weight	1418.24	625.41
Temperature [K]	293(2)	100(2)
Wavelength [Å]	0.71073	0.82657
Crystal system, space group	triclinic, *P*-1	monoclinic, *C*2
Unit cell dimensions [Å] and [°]	*a* = 10.0417(15) *α* = 90.796(11)	*a* = 28.5592(13)
	*b* = 11.1083(15) *β* = 99.044(12)	*b* = 7.5327(4) *β* = 96.751(5)
	*c* = 12.5958(17) *γ* = 95.502(12)	*c* = 11.9908(7)
Volume [Å^3^]	1380.6(3)	2561.7(2)
*Z*, Calculated density [Mg⋅m^−3^]	1, 1.706	4, 1.622
Absorption coefficient [mm^−1^]	2.336	2.932
F(000)	715	1268
Crystal size [mm]	0.400 × 0.360 × 0.190	0.370 × 0.110 × 0.050
Theta range for data collection [°]	2.064 to 26.372	4.086 to 31.102
Limiting indices	−10 <= *h* <= 12	−35 <= *h* <= 35
	−13 <= *k* <= 13	−9 <= *k* <= 9
	−15 <= *l* <= 15	−14 <= *l* <= 14
Reflections collected/unique	9252/9252 [*R*(int) = 0.0637]	17143/5140 [*R*(int) = 0.0247]
Completeness [%] to theta [°]	25.242° 99.8%	29.732° 97.5%
Absorption correction	Analytical	Numerical
Max. and min. transmission	0.665 and 0.455	0.867 and 0.410
Refinement method	Full-matrix least-squares on F^2^	Full-matrix least-squares on F^2^
Data/restraints/parameters	9252/6/332	5140/1/325
Goodness-of-fit on *F*^2^	1.011	1.063
Final *R* Indices [*I* > 2sigma(*I*)]	*R*_1_^a^ = 0.0913, w*R*_2_^b^ = 0.2373	*R*_1_^a^ = 0.0251, w*R*_2_^b^ = 0.0684
*R* indices (all data)	*R*_1_^a^ = 0.1205, w*R*_2_^b^ = 0.2497	*R*_1_^a^ = 0.0255, w*R*_2_^b^ = 0.0686
Absolute structure parameter	N/D	0.007(2)
Largest diff. peak and hole [eÅ^−3^]	2.320 and −1.326	0.534 and −0.361

^a^ R1 = Σ||*F*_0_| − |*F_c_*||/Σ|*F*_0_|; ^b^ wR2 = [Σw(*F*_0_^2^ − *F_c_*^2^)^2^/Σ(w(*F*_0_^2^)^2^)]^1/2^.

## Supplementary Materials

Supplementary materials can be found at https://www.mdpi.com/1422-0067/21/13/4587/s1. Figure S1: ^1^H NMR spectrum of ligand **HL2** (700 MHZ, CDCl_3_)., Figure S2: ^13^C NMR spectrum of ligand **HL2** (700 MHZ, CDCl_3_), Figure S3: ^1^H NMR spectrum of ligand **HL1** (400 MHZ, DMSO-_d6_), Figure S4: ^13^C NMR spectrum of ligand **HL1** (400 MHZ, DMSO-_d6_), Figure S5: IR spectrum of ligand **HL1**, Figure S6: IR spectrum of ligand **HL2**, Figure S7: IR spectrum of complex {[Cu_2_(L1)_2_Cl_2_]_2_[CuCl_4_]}·2MeCN **1**, Figure S8: IR spectrum of complex [Cu_2_(L2)Cl_3_]0.5MeCN **2**, Figure S9: TG-DTA traces of {[Cu_2_(L1)_2_Cl_2_]_2_[CuCl_4_]}·2MeCN·2H_2_O **1**, Figure S10: TG-DTA traces of [Cu_2_(L2)Cl_3_]0.5MeCN·**2**, Figure S11: [CuCl_4_]^2−^ unit, acetonitrile and O34 water (hydrogen atoms are missing) molecules of **1** with ellipsoids plotted at 30% probability level, Figure S12: Perspective view of packing of **1** along *a* axis with chains composed of dimers connected by chloride bridges running along this axis and with [CuCl_4_]^2−^ units and acetonitrile molecules located between adjacent *ab* layers, Figure S13: Packing of **1** along a axis with chains composed of dimers connected by chloride bridges running along this axis and with [CuCl_4_]^2−^ units and acetonitrile molecules located between adjacent *ab* layers, Figure S14: Crystal structure of complex **1**. Geometry formed by base-to-edge sharing polyhedra of a square pyramidal doubly chloro bridged dimer and polyhedral representation of complex {[Cu_2_(L1)Cl_2_]_2_[CuCl_4_]}·2MeCN·2H_2_O **1** with SP-1 to show the resemblance, Figure S15: The X-band powder EPR spectra of complexes **1** and **2** at 77 K, Figure S16: Theoretical prediction of the absorption spectrum of ligand **HL1** absorption spectrum in gas phase (upper panel) and acetonitrile (lower panel) calculated with B3LYP (blue line), CAM-B3LYP (green line) and PBE0 (violet line) functionals (numerical values of absorption wavelengths for most intensive transitions given for PBE0 functional), Figure S17: Frontier orbitals of ligand **HL1**, involved in most intenstive transitions (PBE0/def2-TZVP/acetonitrile) with corresponding wavelengths, Figure S18: Theoretical prediction of the absorption spectrum of ligand **HL2** in gas phase (upper panel) and acetonitrile (lower panel) calculated with B3LYP (blue line), CAM-B3LYP (green line) and PBE0 (violet line) functionals (numerical values of absorption wavelengths for most intensive transitions given for PBE0 functional), Figure S19: Frontier orbitals of ligand **HL2**, involved in most intenstive transitions (PBE0/def2-TZVP/acetonitrile) with corresponding wavelengths, Figure S20: {[Cu_2_(L1)Cl_2_]_2_[CuCl_4_]}·2MeCN·2H_2_O (**1**)/Si, scan size 5 µm, multistage spin coating. Phase images map of layer property including mechanical, chemical, and viscoelastic. Amplitude- the image of height, in which the dimension of *z* axis was reduced, Figure S21: [Cu_2_(L2)Cl_3_] 0.5 MeCN·**2** /PMMA/Si a) height, b) phase, c) amplitude, scan size 5 μm, R_a_ = 5.15-19.9 nm, R_q_ = 3.66-25.9 nm, height 15 nm, 3000 rpm PMMA x1, complex **2** 3000 rpm x2, complex + PMMA 3000 rpm x1, complex 3000 rpm x1, 5s, Table S1: Theoretical chemical shift for ^1^H and ^13^C NMR for ligand **HL1** and ligand **HL2** [ppm] calculated with B3LYP/def2-TZVP approach in the gas phase and solvents., Table S2: The Cu(II) chloro- and oxide- bridge systems, Table S3: Relevant photophysical data of studied compounds, (*λ*_em_, *λ*_ex_ nm, λ[nm] (ε [dm^3^ mol^−1^ cm^−1^ ])), Table S4: Theoretical absorption wavelengths and corresponding oscillator strengths for ligands **HL1** and **HL2** calculated with def2-TZVP basis set in linear response approach.

## References

[1] Bhunia A., Vojtíšek P., Bertolasi V., Manna S.C. (2019). Tridentate Schiff base coordinated trigonal bipyramidal/square pyramidal copper(II) complexes: Synthesis, crystal structure, DFT/TD-DFT calculation, catecholase activity and DNA binding. J. Mol. Struct..

[2] Ahamad M.N., Iman K., Raza K., Kumar M., Ansari A., Ahmad M., Shahid M. (2020). Anticancer properties, apoptosis and catecholase mimic activities of dinuclear cobalt(II) and copper(II) Schiff base complexes. Bioorg. Chem..

[3] Zarei L., Asadi Z., Dusek M., Eigner V. (2019). Homodinuclear Ni (II) and Cu (II) Schiff base complexes derived from O-vanillin with a pyrazole bridge: Preparation, crystal structures, DNA and protein (BSA) binding, DNA cleavage, molecular docking and cytotoxicity study. J. Photochem. Photobiol. A Chem..

[4] Majumder S., Sarkar S., Sasmal S., Sañudo E.C., Mohanta S. (2011). Heterobridged Dinuclear, Tetranuclear, Dinuclear-Based 1-D, and Heptanuclear-Based 1-D Complexes of Copper(II) Derived from a Dinucleating Ligand: Syntheses, Structures, Magnetochemistry, Spectroscopy, and Catecholase Activity. Inorg. Chem..

[5] Barwiolek M., Szlyk E., Kozakiewicz A., Muziol T., Bieńko A., Jezierska J. (2018). X-ray structure, magnetic and fluorescence characteristics of new Cu(II) complexes with Schiff bases derived from 2-(2-aminoethyl)pirydyne and 2-hydroxy-1-naphthaldehyde; morphology and fluorescence of their thin films. Dalton Trans..

[6] Majeste R.J., Klein C.L., Stevens E.D. (1983). Structure of a binuclear copper(II) complex: μ-{2,6-bis[(2-pyridyl)methyliminomethyl]-p-cresolato-N,N′,N′′,N′′′,μ-O}-μ-chloro-dichlorodicopper(II) dihydrate, C_21_H_19_Cl_3_Cu_2_N_4_O·2H2O. Acta Crystallogr. Sect. C Cryst. Struct. Commun..

[7] Žilić D., Rakvin B., Milić D., Pajić D., Đilović I., Cametti M., Dzolic Z. (2014). Crystal structures and magnetic properties of a set of dihalo-bridged oxalamidato copper(ii) dimers. Dalton Trans..

[8] Brown S.J., Tao X., Wark T.A., Stephan D.W., Mascharak P.K. (1988). Synthetic analog approach to metallobleomycins. 4. New halobridged dimeric and polymeric (infinite zigzag chain) complexes of copper(II) with peptide ligands related to bleomycins. Inorg. Chem..

[9] Holm R.H., Kennepohl P., Solomon E.I. (1996). Structural and Functional Aspects of Metal Sites in Biology. Chem. Rev..

[10] Tardito S., Bassanetti I., Bignardi C., Elviri L., Tegoni M., Mucchino C., Bussolati O., Franchi-Gazzola R., Marchiò L. (2011). Copper Binding Agents Acting as Copper Ionophores Lead to Caspase Inhibition and Paraptotic Cell Death in Human Cancer Cells. J. Am. Chem. Soc..

[11] Keypour H., Shayesteh M., Rezaeivala M., Sayin K. (2016). Dinuclear Cu(II) complexes of compartmental Schiff base ligands formed from unsymmetrical tripodal amines of varying arm lengths: Crystal structure of [Cu2L1](ClO4)2 and theoretical studies. J. Mol. Struct..

[12] Gennarini F., David R., López I., Le Mest Y., Reglier M., Belle C., Thibon-Pourret A., Jamet H., Le Poul N. (2017). Influence of Asymmetry on the Redox Properties of Phenoxo- and Hydroxo-Bridged Dicopper Complexes: Spectroelectrochemical and Theoretical Studies. Inorg. Chem..

[13] Holz R.C., Brink J.M., Gobena F.T., O’Connor C.J. (1994). Dinuclear Copper(II) Complexes with Carboxylate-Rich Coordination Environments. Models for Substituted Copper(II) Aminopeptidases. Inorg. Chem..

[14] Bühl M., Ashbrook S.E., Dawson D.M., Doyle R., Hrobarik P., Kaupp M., Smellie I.A. (2016). Paramagnetic NMR of Phenolic Oxime Copper Complexes: A Joint Experimental and Density Functional Study. Chem. Eur. J..

[15] Papatriantafyllopoulou C., Stamatatos T.C., Wernsdorfer W., Teat S.J., Tasiopoulos A.J., Escuer A., Perlepes S.P. (2010). Combining Azide, Carboxylate, and 2-Pyridyloximate Ligands in Transition-Metal Chemistry: Ferromagnetic NiII5Clusters with a Bowtie Skeleton. Inorg. Chem..

[16] Sasmal S., Hazra S., Kundu P., Majumder S., Aliaga-Alcalde N., Ruiz E., Mohanta S. (2010). Magneto-Structural Correlation Studies and Theoretical Calculations of a Unique Family of Single End-to-End Azide-Bridged NiII4Cyclic Clusters. Inorg. Chem..

[17] Manca G., Cano J., Ruiz E. (2009). Exchange Interactions in Azido-Bridged Ligand NiIIComplexes: A Theoretical Analysis. Inorg. Chem..

[18] Mallah T., Kahn O., Gouteron J., Jeannin S., Jeannin Y., O’Connor C.J. (1987). Novel polypyrazolylborate ligands: Coordination control through 3-substituents of the pyrazole ring. Inorg. Chem..

[19] Nayak M., Jana A., Fleck M., Hazra S., Mohanta S. (2010). A unique example of a three component cocrystal of metal complexes. CrystEngComm.

[20] Koval I.A., Gamez P., Belle C., Selmeczi K., Reedijk J. (2006). Synthetic models of the active site of catechol oxidase: Mechanistic studies. Chem. Soc. Rev..

[21] Roznyatovsky V.V., Borisova N.E., Reshetova M.D., Ustynyuk Y.A., Aleksandrov G.G., Eremenko I., Moiseev I.I. (2004). Dinuclear and polynuclear transition metal complexes with macrocyclic ligands. 6. New dinuclear copper(ii) complexes with macrocyclic Schiff bases derived from 4-tert-butyl-2,6-diformylphenol. Russ. Chem. Bull..

[22] Fielden J., Sprott J., Long D.-L., Kögerler P., Cronin L. (2006). Controlling Aggregation of Copper(II)-Based Coordination Compounds: From Mononuclear to Dinuclear, Tetranuclear, and Polymeric Copper Complexes. Inorg. Chem..

[23] Khan S., Sproules S., Natrajan L.S., Harms K., Chattopadhyay S. (2018). End-on cyanate or end-to-end thiocyanate bridged dinuclear copper(ii) complexes with a tridentate Schiff base blocking ligand: Synthesis, structure and magnetic studies. New J. Chem..

[24] Biswas A., Drew M.G., Song Y., Ghosh A. (2011). Effect of anionic co-ligands on structure and magnetic coupling of bis(μ-phenoxo)-bridged dinuclear copper(II) complexes. Inorg. Chim. Acta.

[25] Asokan A., Manoharan P.T. (1999). 1H NMR Studies on Strongly Antiferromagnetically Coupled Dicopper(II) Systems. Inorg. Chem..

[26] Das K., Mondal T.K., Garribba E., Fondo M., Sinha C., Datta A. (2014). Phenoxo bridged tetranuclear copper(II) and dinuclear zinc(II) complexes of 2,6-diformyl-4-methylphenol-di(benzoylhydrazone): Synthesis, structure, spectra and magnetism. Inorg. Chim. Acta.

[27] Grzybowski J.J., Merrell P.H., Urbach F.L. (1978). Dicopper(II) Complexes with Binucleating Ligands Derived from 2-Hydroxy-5-methylisophthaldehyde and 2-(2-aminoethyl)pyridine. Inorg. Chem..

[28] Thakurta S., Roy P., Rosair G., Gómez-García C.J., Garribba E., Mitra S. (2009). Ferromagnetic exchange coupling in a new bis(μ-chloro)-bridged copper(II) Schiff base complex: Synthesis, structure, magnetic properties and catalytic oxidation of cycloalkanes. Polyhedron.

[29] Sorar I., Şener M.K., Tepehan F.Z., Gül A., Koçak M.B. (2008). Structural and optical studies of tetra (tricarbethoxy)-substituted metallophthalocyanines. Thin Solid Films.

[30] Sano T., Nishio Y., Hamada Y., Takahashi H., Usuki T., Shibata K. (2000). The electroluminescence of organic materials. J. Mater. Chem..

[31] Bräuer B., Zahn D.R., Rüffer T., Salvan G. (2006). Deposition of thin films of a transition metal complex by spin coating. Chem. Phys. Lett..

[32] Barwiolek M., Szłyk E., Surdykowski A., Wojtczak A. (2013). New nickel(ii) and copper(ii) complexes with unsymmetrical Schiff bases derived from (1R,2R)(−)cyclohexanediamine and the application of Cu(ii) complexes for hybrid thin layers deposition. Dalton Trans..

[33] Barwiolek M., Szłyk E., Berg A., Wojtczak A., Muziol T.M., Jezierska J. (2014). Structural studies of copper(ii) complexes with 2-(2-aminoethyl)pyridine derived Schiff bases and application as precursors of thin organic–inorganic layers. Dalton Trans..

[34] Gultneh Y., Tesema Y.T., Yisgedu T.B., Butcher R.J., Wang G., Yee G.T. (2006). Studies of a Dinuclear Manganese Complex with Phenoxo and Bis-acetato Bridging in the Mn2(II,II) and Mn2(II,III) States: Coordination Structural Shifts and Oxidation State Control in Bridged Dinuclear Complexes. Inorg. Chem..

[35] Sikdar Y., Modak R., Bose D., Banerjee S., Bieńko D., Zierkiewicz W., Bieńko A., Das Saha K., Goswami S. (2015). Doubly chloro bridged dimeric copper(ii) complex: Magneto-structural correlation and anticancer activity. Dalton Trans..

[36] Manzur J., García A.M., Vega A., Ibañez A. (2007). Synthesis and structure of copper(II) complexes with L–OH (L–OH = 2,6-bis-[N-(2-pyridylethyl)-formidoyl]-4-methyl-phenol). Polyhedron.

[37] Spek A.L. (2009). Structure validation in chemical crystallography. Acta Cryst. Sect. D Boil. Cryst..

[38] Addison A.W., Rao T.N., Reedijk J., van Rinn J., Verschoor G.C. (1984). Synthesis, structure, and spectroscopic properties of copper(II) compounds containing nitrogen-sulphur donor ligands; the crystal and molecular structure of aqua[1,7-bis(N-methylbenzimidazol-2′-yl)-2,6-dithiaheptane]copper(II) perchlorate. J. Chem. Soc. Dalton Trans..

[39] Okuniewski A., Rosiak D., Chojnacki J., Becker B. (2015). Coordination polymers and molecular structures among complexes of mercury(II) halides with selected 1-benzoylthioureas. Polyhedron.

[40] Chilton N.F., Anderson R.P., Turner L.D., Soncini A., Murray K.S. (2013). PHI: A powerful new program for the analysis of anisotropic monomeric and exchange–coupled polynuclear d- and f-block complexes. J. Comput. Chem..

[41] Roundhill S.G.N., Roundhill D.M., Bloomquist D.R., Landee C., Willett R.D., Dooley D.M., Gray H.B. (1979). Molecular structure and magnetic properties of the chloro-bridged dimer chloro[hydrotris(1-pyrazolyl)borato]copper(II). Observation of a ferromagnetic ground state. Inorg. Chem..

[42] Marsh W.E., Hatfield W.E., Hodgson D.J. (1982). Magnetic interactions in chloro-bridged dimers. Structural characterization of aquadichlorobis(2-methylpyridine)copper(II) and bis[dichlorobis(2-methylpyridine)copper(II)]. Inorg. Chem..

[43] Marsh W.E., Patel K.C., Hatfield W.E., Hodgson D.J. (1983). Magnetic interactions in chloro-bridged copper(II) dimers. Structural and magnetic characterization of bis(.mu.-chloro)bis[chloro(N,N,N’-triethylethylenediamine)copper(II)], [Cu(Et3en)Cl2]2. Inorg. Chem..

[44] Crawford V.H., Richardson H.W., Wasson J.R., Hodgson D.J., Hatfield W.E. (1976). Relation between the singlet-triplet splitting and the copper-oxygen-copper bridge angle in hydroxo-bridged copper dimers. Inorg. Chem..

[45] Graham B., Hearn M.T.W., Junk P.C., Kepert C.M., Mabbs F.E., Moubaraki B., Murray K.S., Spiccia L. (2001). Syntheses, Crystal Structures, Magnetic Properties, and EPR Spectra of Tetranuclear Copper(II) Complexes Featuring Pairs of “Roof-Shaped” Cu_2_X_2_ Dimers with Hydroxide, Methoxide, and Azide Bridges. Inorg. Chem..

[46] Skorda K., Stamatatos T.C., Vafiadis A.P., Lithoxoidou A.T., Terzis A., Perlepes S.P., Mrozinski J., Raptopoulou C.P., Plakatouras J.C., Bakalbassis E.G. (2005). Copper(II) chloride/1-methylbenzotriazole chemistry: Influence of various synthetic parameters on the product identity, structural and magnetic characterization, and quantum-chemical studies. Inorg. Chim. Acta.

[47] Grove H., Julve M., Sletten J., Lloret F. (2001). Solid state polymerization causing transition to a ferromagnetic state. Crystal structures and magnetic properties of [Cu2(dpp)(H2O)(dmso)Cl4]·dmso and [Cu2(dpp)Cl4]n (dpp = 2,3-bis(2-pyridyl)pyrazine). J. Chem. Soc. Dalton Trans..

[48] Rodríguez M., Llobet A., Corbella C.M. (2000). A theoretical analysis of how geometrical distortions on Cu(μ-Cl)2Cu dimers influence their electronic and magnetic properties. Polyhedron.

[49] Kozlevčar B., Šegedin P. (2008). Structural Analysis of a Series of Copper(II) Coordination Compounds and Correlation with their Magnetic Properties. Croat. Chem. Acta.

[50] Brudenell S.J., Spiccia L., Tiekink E.R.T. (1996). Binuclear Copper(II) Complexes of Bis(pentadentate) Ligands Derived from Alkyl-Bridged Bis(1,4,7-triazacyclonane) Macrocycles. Inorg. Chem..

[51] Lever A.B.P. (1968). Studies in Physical and Theoretical Chemistry 33: Inorganic Electronic Spectroscopy.

[52] Boudreaux E.A., Mulay L.N. (1976). Theory and Applications of Molecular Paramagnetism.

[53] Oxford Diffraction Ltd. (2000). CrysAlis RED and CrysAlis CCD.

[54] Kabsch W. (2010). XDS. Acta Cryst..

[55] Krug M., Weiss M.S., Heinemann U., Mueller U. (2012). XDSAPP: A graphical user interface for the convenient processing of diffraction data using XDS. J. Appl. Cryst..

[56] Sheldrick G.M. (2015). Crystal structure refinement with SHELXL. Acta Cryst. Sect. C Struct. Chem..

[57] Brandenburg K. (2001). DIAMOND, Crystal Impact GbR.

[58] Farrugia L.J. (2012). WinGX and ORTEP for Windows: An update. J. Appl. Cryst..

[59] Frisch M.J., Trucks G.W., Schlegel H.B., Scuseria G.E., Robb M.A., Cheeseman J.R., Scalmani G., Barone V., Petersson G.A., Nakatsuji H. (2016). Gaussian 16.

[60] Zieliński W., Rajca A. (1995). Metody Spektroskopowe i ich Zastosowanie do Identyfikacji Zwiazków Organicznych.

[61] Mueller U., Forster R., Hellmig M., Huschmann F.U., Kastner A., Malecki P., Pühringer S., Röwer M., Sparta K., Steffien M. (2015). The macromolecular crystallography beamlines at BESSY II of the Helmholtz-Zentrum Berlin: Current status and perspectives. Eur. Phys. J. Plus.

