# Supplementary material for: Dinuclear Copper(II) Complexes with Schiff Bases Derived from 2-Hydroxy-5-Methylisophthalaldehyde and Histamine or 2-(2-Aminoethyl)pyridine and Their Application as Magnetic and Fluorescent Materials in Thin Film Deposition
Source: Int J Mol Sci. 2020 Jun 28;21(13):4587. doi: 10.3390/ijms21134587 (PMC7370049; doi:10.3390/ijms21134587)
Supplement: Supplementary file 1 [file ijms-21-04587-s001.pdf]

International Journal of
Molecular Sciences

MDPI

Supplementary Materials

Dinuclear copper(II) complexes with Schiff bases derived from 2-hydroxy-5-methylisophthalaldehyde and histamine or 2-(2-aminoethyl)pyridine and their application as magnetic and fluorescent materials in thin film deposition

Magdalena Barwiolek[1],* Anna Kaczmarek-Kędziera[1], * Tadeusz M. Muziol[1], Dominika Jankowska[1], Julia Jezierska[2] and Alina Bieńko[2]

[1] Faculty of Chemistry, Nicolaus Copernicus University in Torun, 87-100 Torun, Poland, e-mail: mbarwiolek@umk.pl (M.B.); eoadk@chem.umk.pl (A.K.K.); tadeuszmuziol@wp.pl (T.M.M); (D.J); 278998@stud.umk.pl

[2] Faculty of Chemistry, University of Wroclaw, 14 Joliot-Curie, 50-383 Wroclaw, Poland julia.jezierska@chem.uni.wroc.pl (J.J); alina.bienko@uwr.edu.pl (A.B.)

* Correspondence: mbarwiolek@umk.pl; Tel.: +48-56-611-4516 (M.B.); eoadk@chem.umk.pl

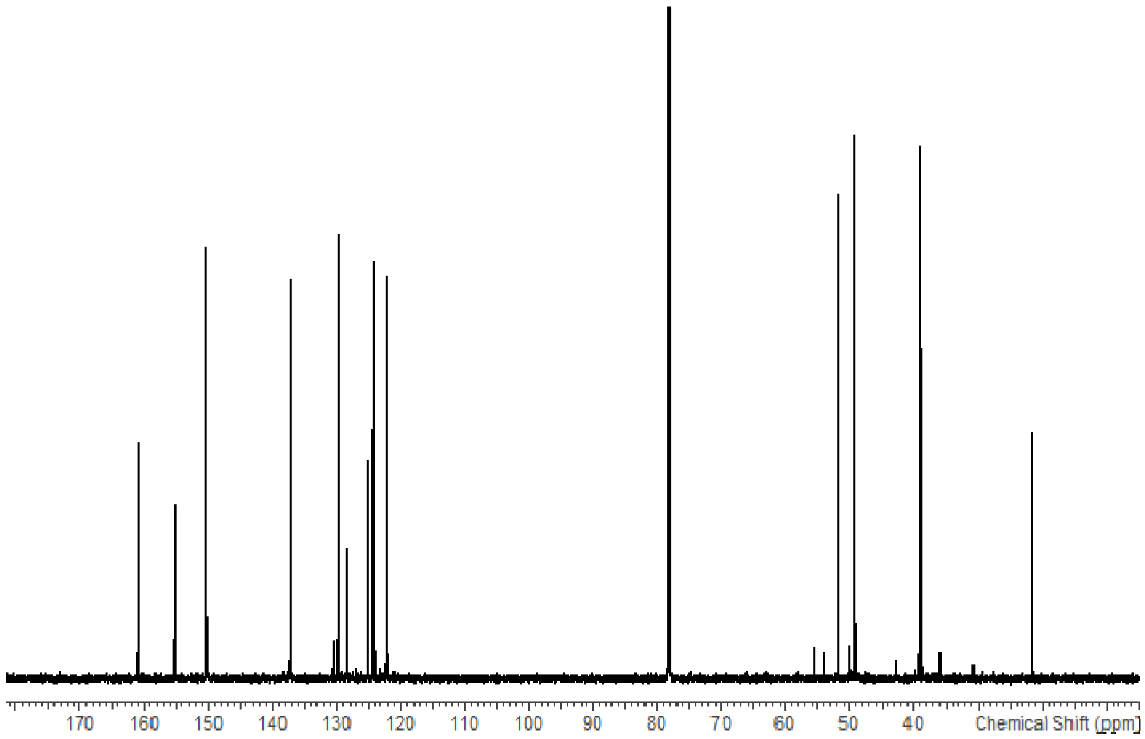

Figure S1. ^{1}H NMR spectrum of ligand **HL2** (700 MHZ, $CDCl_3$).

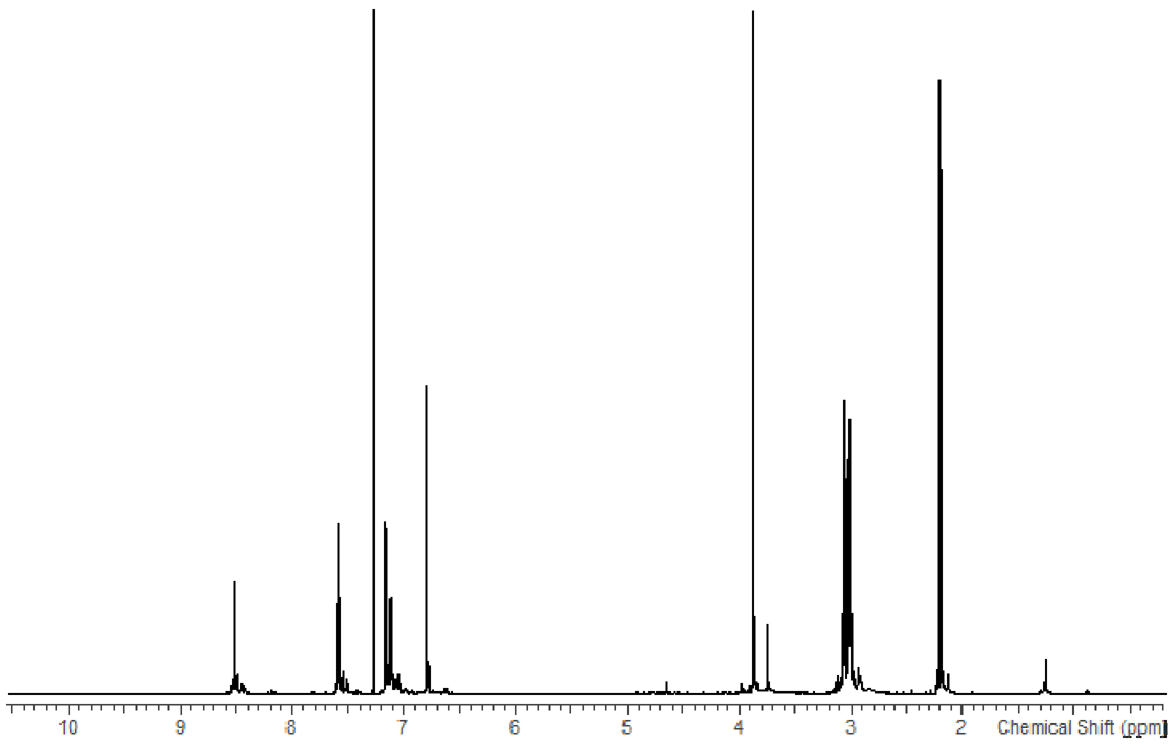

Figure S2. ^{13}C NMR spectrum of ligand **HL2** (700 MHZ, $CDCl_3$).

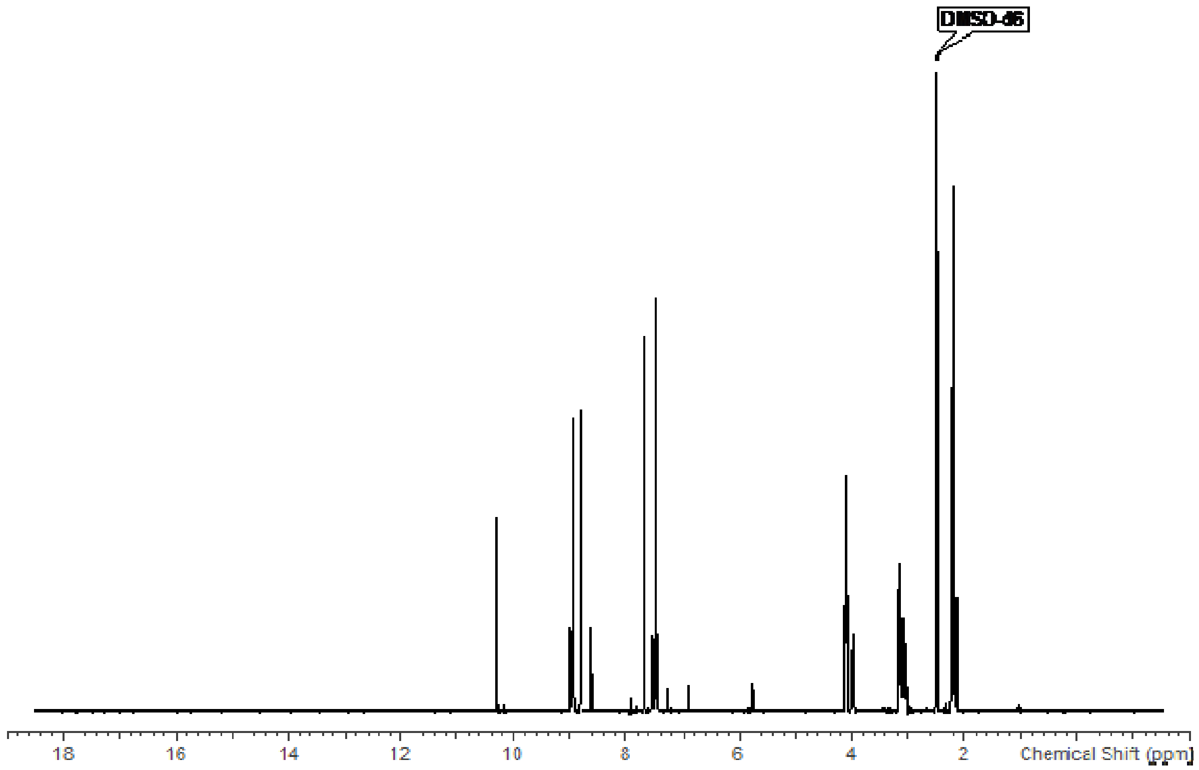

Figure S3. ^{1}H NMR spectrum of ligand **HL1** (400 MHZ, DMSO-d_6).

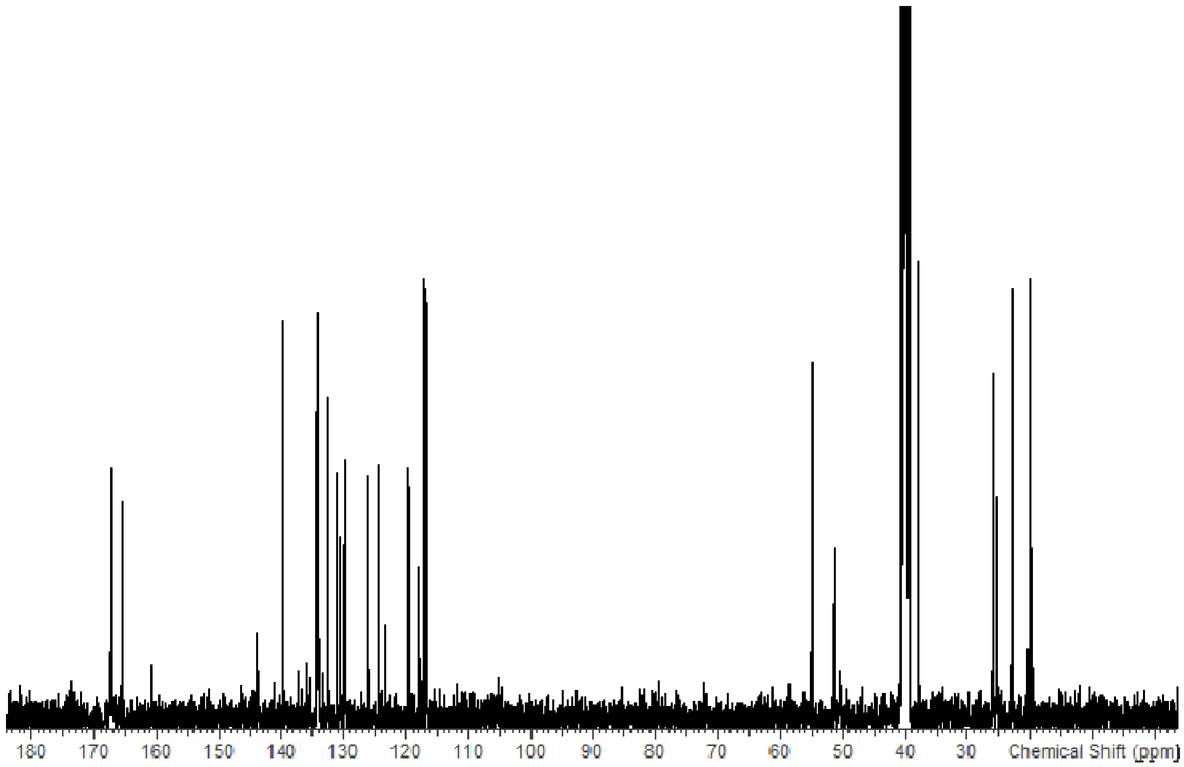

Figure S4. ^{13}C NMR spectrum of ligand **HL1** (400 MHZ, DMSO-$_{d6}$). .

Table S1. Theoretical chemical shift for ^{1}H and ^{13}C NMR for ligand **HL1** and ligand **HL2** [ppm] calculated with B3LYP/def2-TZVP approach in the gas phase and solvents.

**Ligand1**	**Experimental**			Theoretical		
**^{1}H [ppm]**	**σ**			Gas phase	ACN	DMSO
	2.21	(s, 3H)	CH_3	2.03,2.55,2.57	2.57,2.14,2.56	2.16,2.56,2.57
	4.10	(m, 2H)	CH_2	3.50,4.47<br>3.53,4.16	3.60,4.36<br>3.65,4.09	3.61,4.35<br>3.65,4.08
	3.16	(m, 2H)	CH_2	2.74,3.16<br>2.86,2.97	2.51,3.23<br>2.60,3.15	2.51,3.24<br>2.59,3.15
	8.48	(s, 1H)	-N=CH-	8.74, 8.74	8.82,8.86	8.82,8.86
	10.32	(s, 1H)	OH	14.01	14.51	14.55
H12	7.69	(s, 1H)	Ar-H	7.54,7.21	7.50,7.71	7.50,7.72
H3	7.48	(m, 1H)	Ar-H	7.58,7.33	7.25,7.32	7.26,7.32
H10	7.29	(m, 1H)	Ar-H	7.04,8.30	7.48,7.48	7.48,7.49
	5.75	(s, 1H)	NH			
**^{13}C[ppm]**	σ			Gas phase	ACN	DMSO
	**20.07**	C1		22.27	130.52	130.51
	**51.43**	**C7**		70.62,70.76	71.19,75.77	71.20,75.82
	**37.83**	**C8**		36.22,37.84	37.26,38.22	37.24,38.24
CHN	**167.14**	**C6**		163.63,170.43	169.37,173.08	169.46,173.12
	**134.45**	**C12**		133.40,137.29	137.92,138.79	138.00,138.83
	**119.59**	**C10**		114.10,120.02	117.10,117.66	117.19,117.74
	**117.07**	C4		125.44,131.49	126.37,130.52	126.38,130.51
	**139.76**	**C2**		131.62	135.13	135.22
	**132.50**	**C3**		139.66,145.78	142.22,147.92	142.27,147.96
	**160.54**	**C5**		170.23	169.75	169.74
	**134.19**	**C9**		150.86,150.66	150.74,151.92	150.76,151.91
**Ligand2**	Experimental			Theoretical		
**^{1}H [ppm]**	σ			Gas phase	ACN	DMSO
	2.21	(s, 3H)	CH_3	1.93,2.40,2.44	2.42,2.04,2.44	2.42,.2.04,2.44
	3.04	(m, 2H)	CH_2	3.51,3.53<br>4.56,4.79	3.55,4.60<br>3.81,4.45	3.56,4.60<br>3.81,4.44
	3.88	(m, 2H)	CH_2	3.25,3.43	3.37,3.41	3.36,3.41

				3.42,3.20	2.82,3.50	2.82,3.50
	8.54	(s, 1H)	–N=CH-	8.19,8.75	8.31,8.85	8.31,8.86
	8.45	(s, 1H)	–OH	13.64	14.20	14.24
	6.61	(s, 1H)	Ar-H	7.13,7.24	7.40,7.43	7.41,7.44
	7.15	(m, 1H)	Ar-H	7.03,7.92	7.64,7.85	7.65,7.86
	7.19	(m, 1H)	Ar-H	7.33,8.01	7.98,8.14	7.98,8.14
	7.61	(m, 1H)	Ar-H	6.98,7.31	7.36,7.60	7.36,7.60
ArHpyalfa	8.49	(broad signal)	OH	6.98,8.98	8.77,9.01	8.77,9.01
**^{13}C[ppm]**	σ			Gas phase	ACN	DMSO
	21.42	C1		22.12	22.28	22.28
	49.36	C7		62.89,71.67	69.46,73.54	69.45,73.53
	51.70	C8		45.04,45.80	46.72,47,68	46.75,47.68
	161.03	C6		165.69,171.42	169.65,173.59	169.74,173.62
	122.30	C12		124.59,125.09	127.12,127.54	127.16,127.57
	124.31	C10		125.18,130.02	129.05,129.44	129.12,129.51
	125.27	C4		124.70,131.25	126.16,129.98	126.17,129.96
	128.45	C2		131.95	135.11	135.19
	129.59	C3		139.60,146.30	142.22,147.84	142.37,147.88
	137.39	C11		139.86,141.59	143.41,143.85	143.47,143.90
	150.27	C13		155.70,156.12	156.62,156.92	156.62,156.90
	155.15	C5		170.02	169.70	169.69
	161.03	C9		168.72,170.79	172.29	172.30

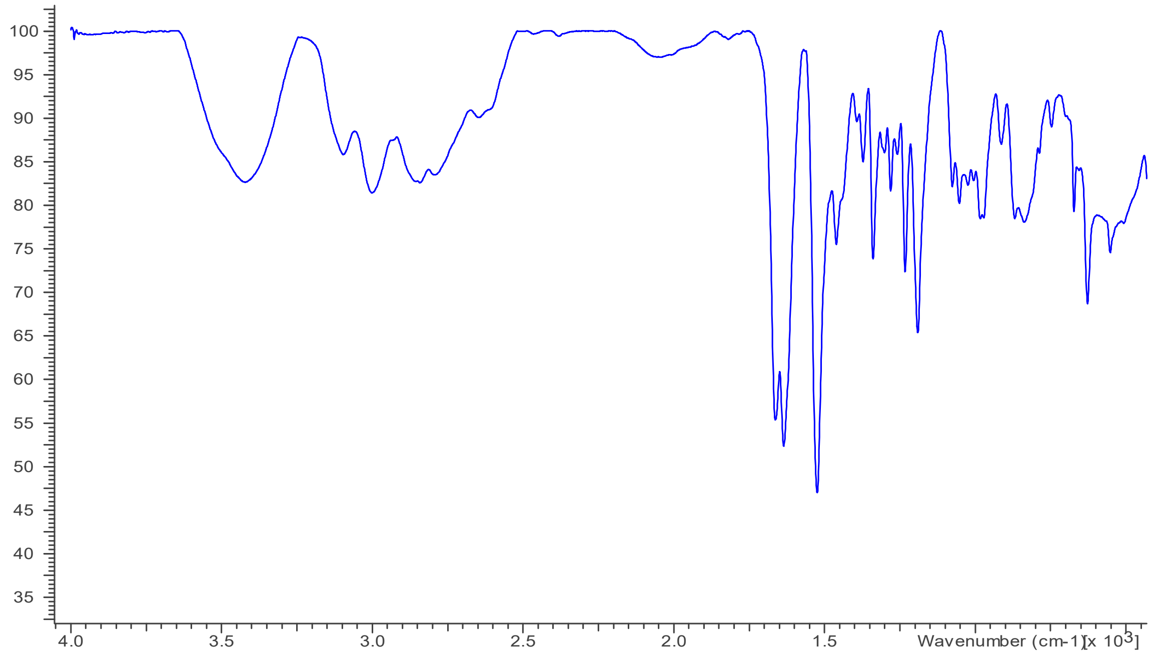

Figure S5. IR spectrum of ligand **HL1.**

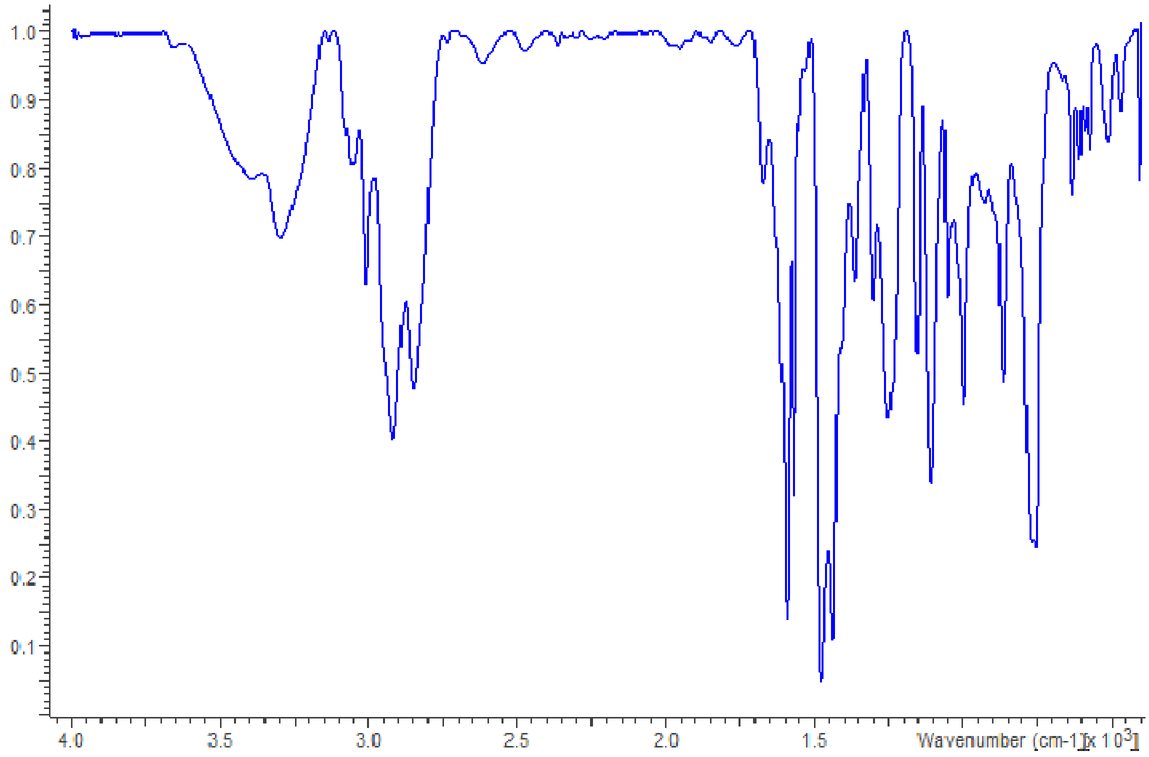

Figure S6. IR spectrum of ligand **HL2.**

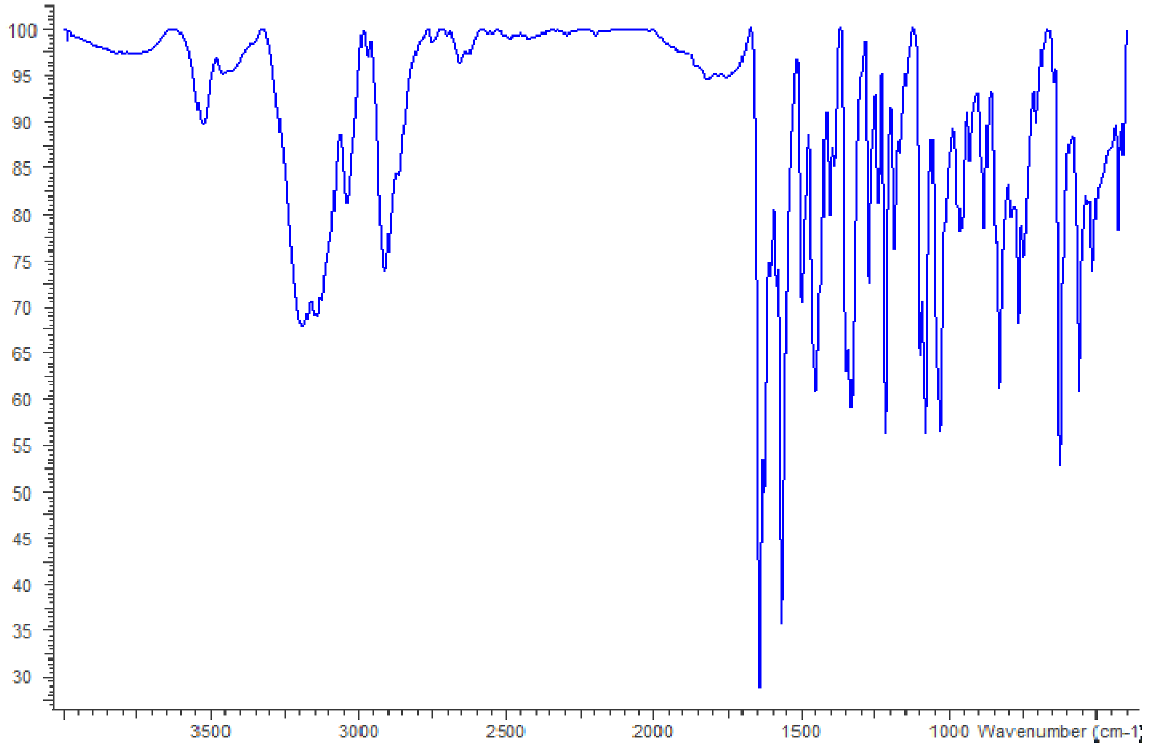

Figure S7. IR spectrum of complex $\{[Cu_2(L1)_2Cl_2]_2[CuCl_4]\}\cdot 2MeCN$ **1.**

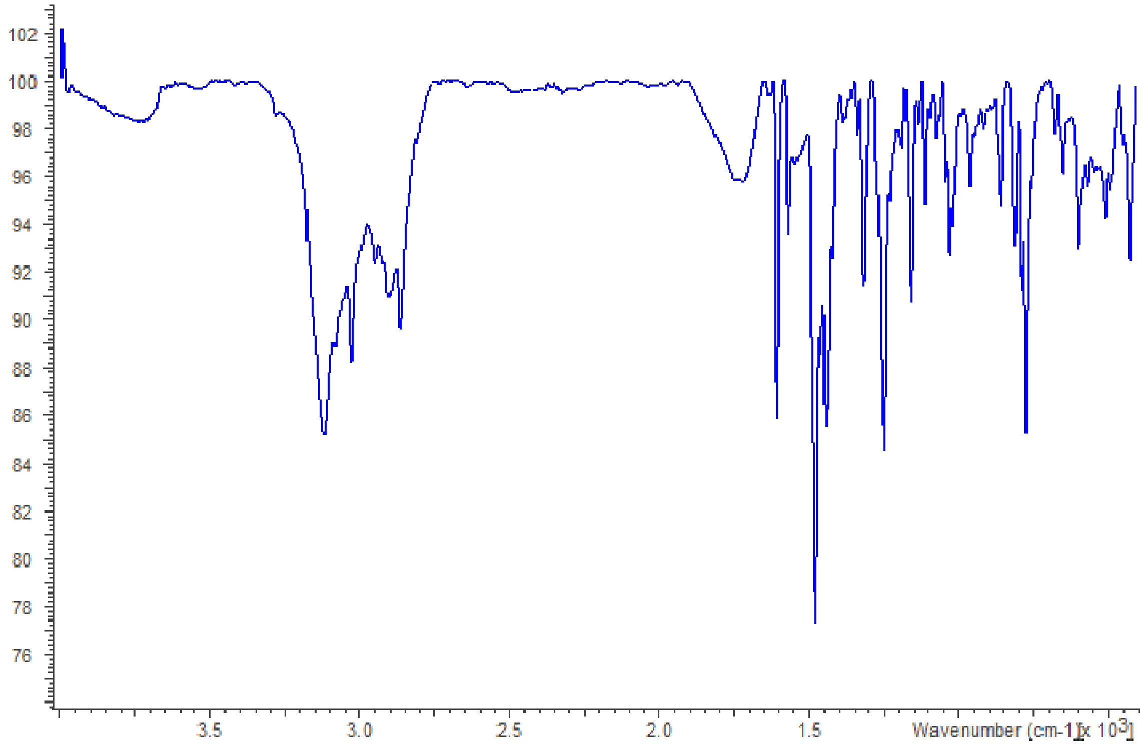

Figure S8. IR spectrum of complex $[Cu_2(L2)Cl_3]0.5MeCN$ **2.**

Thermal analysis

For all the complexes, thermal decomposition studies were performed in the presence of air. The final decomposition product was mixture of copper and copper oxide.

The thermogram of $\{[Cu_2(L1)_2Cl_2]_2[CuCl_4]\}\cdot 2MeCN\cdot 2H_2O$ **1** (Figure S9) presents four DTG peaks with maxima at 206, 325, 428, and 577 °C, which reveal two stages on a TG curve, with 2.3, 19.10, and 66.60% sample mass loss. The partial mass loss in the first step corresponds to water molecules detachment; the second one is connected with $CuCl_4$ and acetonitrile detachment (cal. 17.42%). Further mass reduction in the sample results from the remaining molecules decomposition (found 70.82%; cal. 71.4%) and copper oxide formation at 820 °C.

The $[Cu_2(L2)Cl_3]0.5MeCN\cdot 2$ thermogram reveals two decomposition peaks on the DTG curve. The first peak can be assigned to 1.5 Cl_2, and acetonitrile molecules detachment (exp. 24.69%, cal. 24.4%), (Figure S10). The second DTG peak corresponds to 58.54% mass loss, which can be related to the remaining molecules decomposition followed by CuO formation at 880 °C. The first exothermic peak appeared at 197 °C, the next two - at 444°C, 534°C, and the final one - at 640 °C.

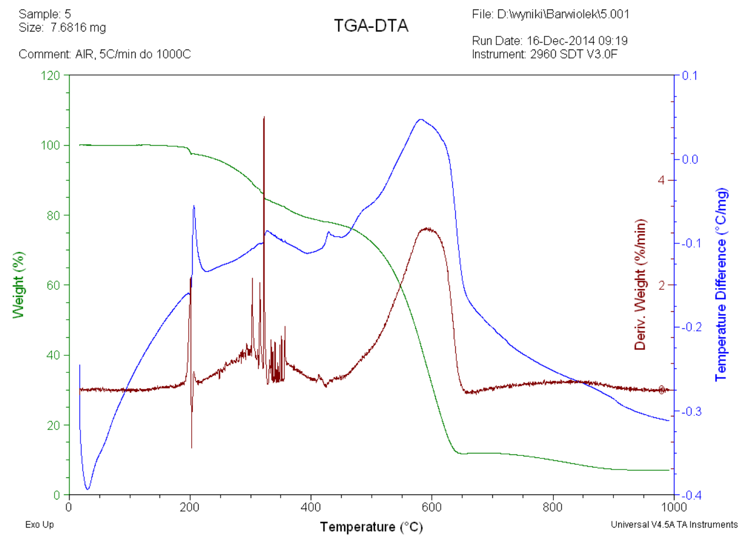

Figure S9. TG-DTA traces of $\{[Cu_2(L1)_2Cl_2]_2[CuCl_4]\}{\cdot}2MeCN{\cdot}2H_2O$ **1**.

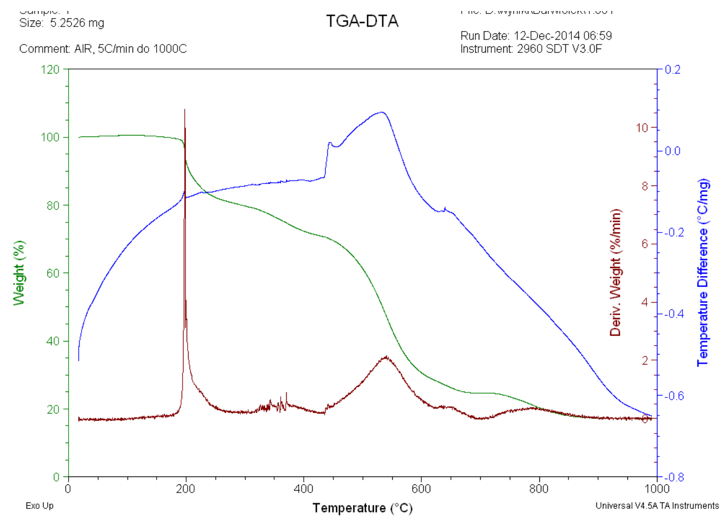

Figure 10. TG-DTA traces of $[Cu_2(L2)Cl_3]0.5MeCN{\cdot}$**2**.

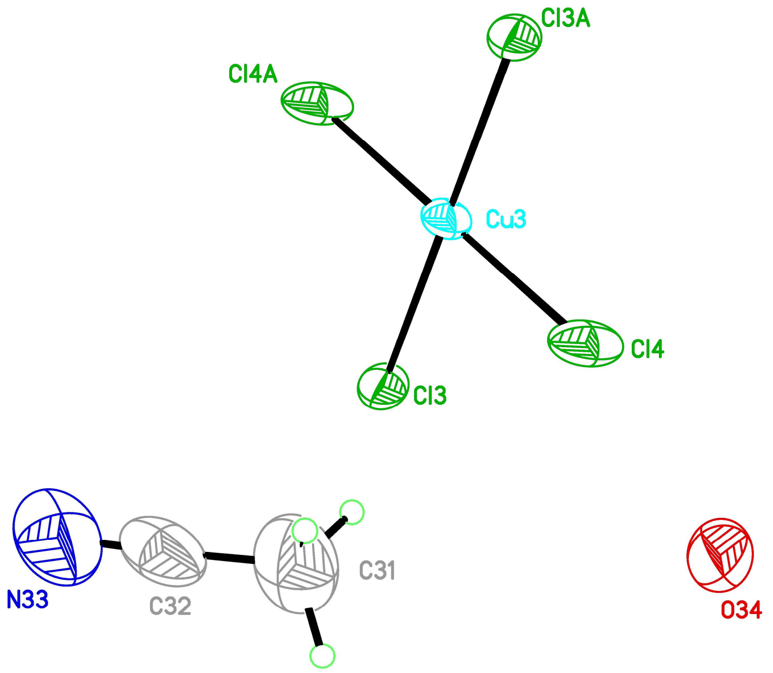

Figure S11. $[CuCl_4]^{2-}$ unit, acetonitrile and O34 water (hydrogen atoms are missing) molecules of **1** with ellipsoids plotted at 30% probability level.

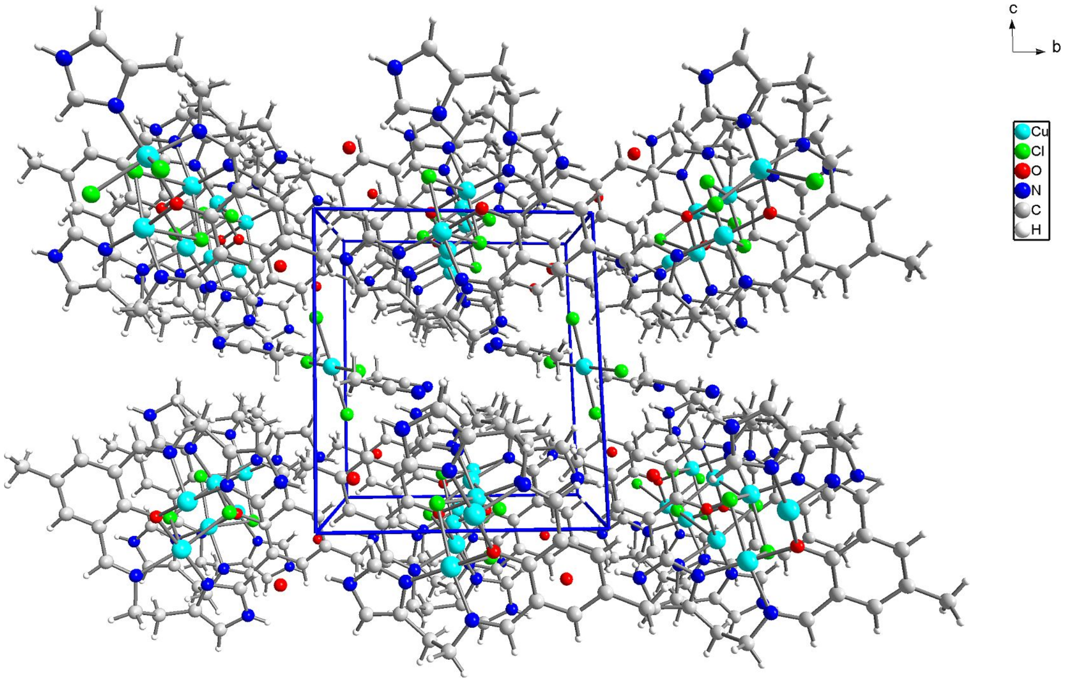

Figure S12. Perspective view of packing of **1** along *a* axis with chains composed of dimers connected by chloride bridges running along this axis and with $[CuCl_4]^{2-}$ units and acetonitrile molecules located between adjacent *ab* layers.

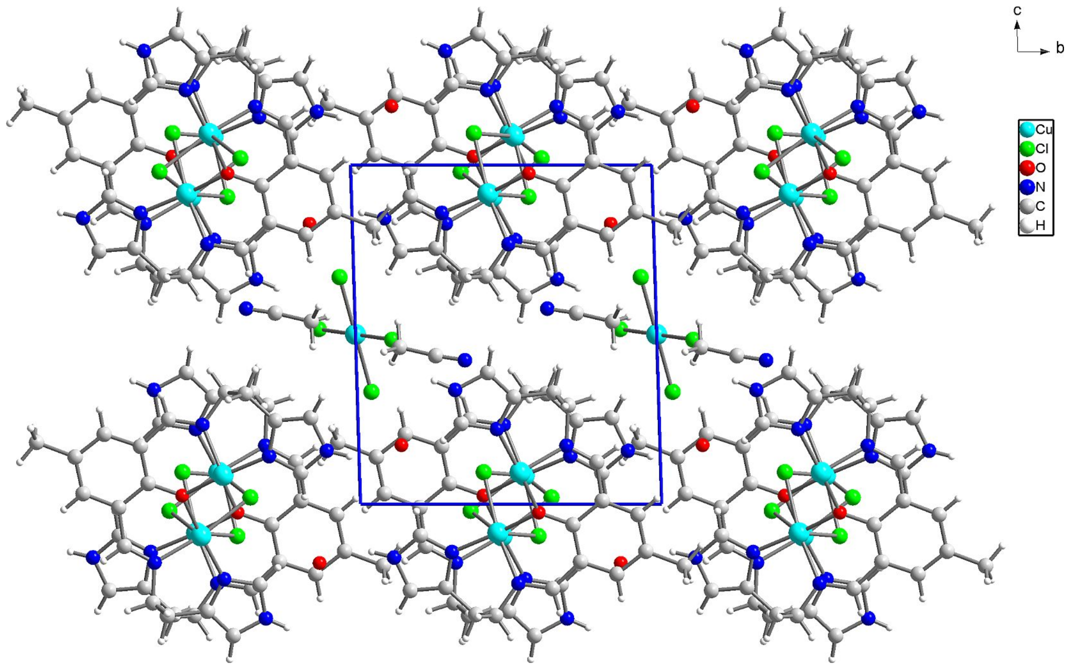

Figure 13. Packing of **1** along *a* axis with chains composed of dimers connected by chloride bridges running along this axis and with $[CuCl_4]^{2-}$ units and acetonitrile molecules located between adjacent *ab* layers.

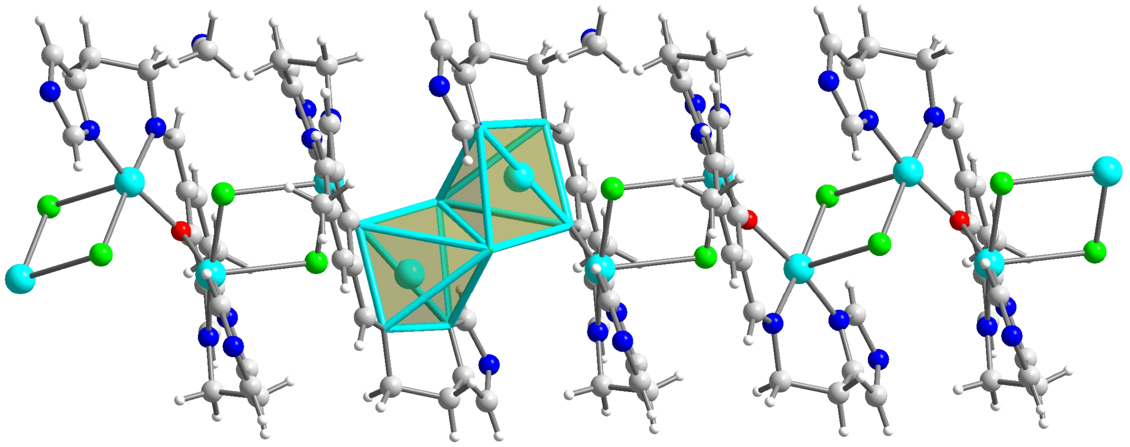

Figure S14. Crystal structure of complex **1**. Geometry formed by base-to-edge sharing polyhedra of a square pyramidal doubly chloro bridged dimer and polyhedral representation of complex $\{[Cu_2(L1)Cl_2]_2[CuCl_4]\}\cdot 2MeCN\cdot 2H_2O$ **1** with SP-1 to show the resemblance.

Table S2. The Cu(II) chloro- and oxide- bridge systems.

Complex	Cu–O–Cu Φ [°]	Cu···Cu [Å]	Cu–O/ [Å]	δ^a [°]/	J $[cm^{-1}]$	Ref.
$[Cu_4L_1(\mu_2\text{-}OH)_2](ClO_4)_4$	197.90<br>95.70	2.939	1.961<br>1.975	159	-27	[1]
$[Cu_2(Me_6tacn)_2(\mu_2\text{-}OH)_2](ClO_4)_2$	100.1	2.971	1.939	180	-45	[2]
$[Cu_2L_2(\mu_2\text{-}OH)_2](ClO_4)_2$	102.48	2.983	1.911<br>1.914	177.6	-186	[3]
$[Cu_2(C_6H_{11}NH_2)_4(\mu_2\text{-}OH)_2](ClO_4)_4$	96.6	2.934	1.960	148	-128	[4]

$[Cu_2(CH_3NH_2)_4(\mu_2\text{-}OH)_2](SO_4)\ H_2O$	99.7<br>91.7<br>88.6	2.782	1.923<br>1.99<br>1.94	133	-4	[5]
$[Cu_2L_3(\mu_2\text{-}OH)_2](BPh_4)_2$	99.60	2.9464	1.931	173	-80	[6]
Complex	Cu–Cl–Cu Φ [°]	Cu···Cu [Å]	Cu-Cl [Å]	φ/R [° $Å^{-1}$]	J [cm^{-1}]	Ref.
$[Cu_2(dpt)_2Cl_2]\ (Cl)_2$	91.42	3.551	2.545	35.91	+85.9	[7]
$[Cu(HL)Cl]_2{\cdot}H_2O$	84.66	3.337	2.668	31.73	+43.2	[8]
$[CuCl_2(PyTn)]_2$	88.60	3.612	2.906	30.49	+27.7	[9]
$[Cu(bpdio)Cl_2]_2$	96.68	3.842	2.844	33.99	+4.87	[10]
$[Cu(Hfsaaep)Cl]_2$	95.27	3.445	2.846	33.6	+0.30	[11]
$C_{22}H_{32}Cl_4Cu_2N_4O_2$	92.65	3.708	2.827	32.77	−0.47	[12]
$[Cu(4\text{-}Metz)(DMF)Cl_2]_2$	95.30	3.386	2.724	34.99	−3.60	[13]
$[Cu(guaH)Cl_3]_2$	95.34	3.570	2.447	37.49	−38.80	[14]
Complex	Cu–O–Cu<br>Cu–Cl–Cu<br>Φ [°]	Cu···Cu [Å]	Cu-O<br>Cu-Cl<br>[Å]	δ [°]/<br>φ/R<br>[° $Å^{-1}$]	J [cm^{-1}]	Ref.
$\{[Cu_2(L1)Cl_2]_2[CuCl_4]\}{\cdot}2MeCN{\cdot}2H_2O$	107.69<br>92.99	3.161<br>3.787	1.956<br>2.344<br>2.786	δ 58.20<br>39.67<br>33.38	−0.31<br>−0.38	This work
$[Cu_2Cl_3(L)]{\cdot}0.5MeCN$ 2	106.26<br>Cu-O-Cu<br>81.31<br>Cu-Cl-Cu	3.163	2.057<br>1.894<br>2.332<br>2.517	δ 150.98<br><br>34.87<br>32.30	−137	This work

L_1=(1,4,7-triazacyclonon-1-ylmethyl)benzene, L_2=N,N,N′,N′′,N′′-pentamethyldiethylenetriamine, L_3 = 1,3-bis(1,4,7-triazacyclonon-1-ylmethyl)benzene; dpt, dipropylenetriamine; HL, 2-((E)-(2-hydroxyethylimino)methyl)-4-bromophenol; pytn, 2-(pyrazol-1-yl)-2-thiazolin; bpdio, 2,2-bis-(2pyridyl)-l,3-dioxolane]; Hfsaaep, 3-[N-2-(pyridylethyl)formimidoyl]-salicylic acid; 4-Metz, 4-methylthiazole; DMF, N,N-dimethylformamide; guaH, guanine.

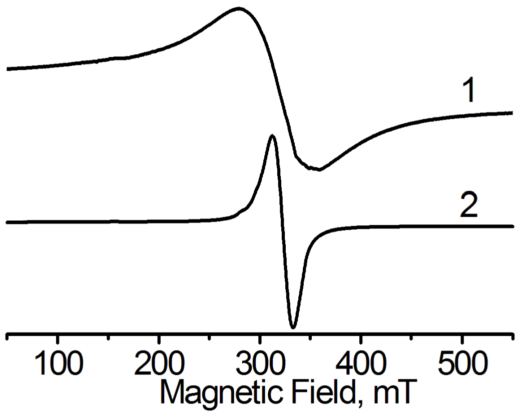

Figure S15. The X-band powder EPR spectra of complexes **1** and **2** at 77 K.

Table S3. Relevant photophysical data of studied compounds, (λ_{em}, λ_{ex} nm, λ[nm] (ε [dm³ mol⁻¹ cm⁻¹])).

Compound	Solvent	λ_{ex} [nm]	λ_{em} [nm]	λ[nm] (ε [dm^3 mol^{-1} cm^{-1}])
**HL1**	acetonitrile	296<br>422	497<br>498	250 (10180)<br>325 (2460)<br>450 (6970)
	chloroform	296<br>422	506<br>503	245 (4720)<br>352 (1569)<br>455 (1290)
	methanol	296<br>422	497<br>500	254 (7116)<br>334 (2056)<br>454 (4522)
**HL2**	acetonitrile	296<br>422	481<br>498	254 (27400)<br>290 (20110)<br>315 (21830)
	chloroform	296<br>422	492<br>471	255 (5400)<br>290 (3570)<br>335 (2310)
	methanol	296<br>422	470<br>496	262 (11134)<br>290 (9448)<br>342 (4426)
**1**	acetonitrile	450	509	270 (12300)<br>365 (26050)<br>455 (8990)<br>650(1410)
	chloroform	450	497	320 (1060)<br>395 (730)<br>575(240)
	methanol	450	504	272 (12300)<br>372 (5800)<br>666(165)
**2**	acetonitrile	450	493	245 (25610)<br>310 (14560)<br>395 (5880)<br>500 (910)
	chloroform	450	489	250 (19070)<br>315 (12080)<br>400 (5150)<br>575(240)<br>665(120)
	methanol	450	500	248 (9836)<br>312 (9007)<br>402 (8511)<br>546(342)<br>626(271)

Table S4. Theoretical absorption wavelengths and corresponding oscillator strengths for ligands **HL1** and **HL2** calculated with def2-TZVP basis set in linear response approach.

**Medium**	**B3LYP**			CAM-B3LYP		PBE0	
**Ligand1**	**λ**	**f**	**Orbital contributions**	λ	f	λ	f
Gas phase	339	0.07	HOMO→LUMO 82%	306	0.19	**326**	**0.14**
	328	0.07	HOMO-1→LUMO 79%			**247**	**0.32**
	254	0.31	HOMO-4→LUMO 83%	232	0.48	**236**	**0.23**
	236	0.37	HOMO-4→LUMO+178%	221	0.39	**229**	**0.24**
	202	0.16	HOMO→LUMO+686%	195	0.17	**200**	**0.21**
Acetonitrile	331	0.17	HOMO→LUMO 92%	303	0.22	**322**	**0.19**
	254	0.26	HOMO-4→LUMO 38%	234	0.54	**246**	**0.41**
	253	0.14	HOMO-5→LUMO 35%<br>HOMO-4→LUMO 34%	228	0.53	**236**	**0.53**
	242	0.55	HOMO-4→LUMO+169%				
	200	0.23	HOMO-2→LUMO+251%	194	0.13	**199**	**0.12**
Dimethylsulfoxide	331	0.18	HOMO→LUMO 92%	303	0.23	**322**	**0.20**
	254	0.35	HOMO-4→LUMO 52%	234	0.56	**246**	**0.42**
	242	0.56	HOMO-4→LUMO+169%			**236**	**0.54**

	200	0.24	HOMO-2→LUMO+257%	196	0.09	**199**	**0.12**
	199	0.30	HOMO→LUMO+445%	195	0.18	**198**	**0.06**
**Ligand2**							
Gas phase	334	0.14	HOMO→LUMO 94%	307	0.19	**325**	**0.15**
	257	0.21	HOMO-4→LUMO 34%	232	0.57	**252**	**0.17**
	255	0.11	HOMO→LUMO+328%	226	0.08	**248**	**0.09**
	240	0.23	HOMO-2→LUMO+149%	221	0.36	**246**	**0.13**
						**234**	**0.32**
	200	0.40	HOMO→LUMO+6 55%	187	0.17	**194**	**0.35**
Acetonitrile	330	0.17	HOMO→LUMO 94%	303	0.22	**321**	**0.19**
	260	0.22	HOMO-1→LUMO 49%	233	0.48	**253**	**0.30**
						**251**	**0.10**
	249	0.18	HOMO-4→LUMO 37%	226	0.10	**244**	**0.24**
	244	0.18	HOMO-6→LUMO 28%<br>HOMO-4→LUMO 25%<br>HOMO-1→LUMO+125%	225	0.44	**239**	**0.15**
	231	0.12	HOMO-1→LUMO+222%	200	0.14	**227**	**0.14**
	203	0.11	HOMO-3→LUMO+4 36%	185	0.11	**193**	**0.54**
Dimethylsulfoxide	330	0.18	HOMO→LUMO 94%	304	0.23	**322**	**0.19**
	260	0.22	HOMO-1→LUMO 46%	234	0.50	**254**	**0.34**
	249	0.18	HOMO-4→LUMO 38%	228	0.29	**251**	**0.10**
	244	0.16	HOMO-6→LUMO 31%	227	0.10	**244**	**0.26**
				225	0.44	**243**	**0.14**
	232	0.11	HOMO-1→LUMO+219%	200	0.14	**227**	**0.14**
	203	0.11	HOMO-10→ LUMO 27%<br>HOMO-3→ LUMO+4 32%	185	0.16	**193**	**0.55**

Ligand absorption spectrum:

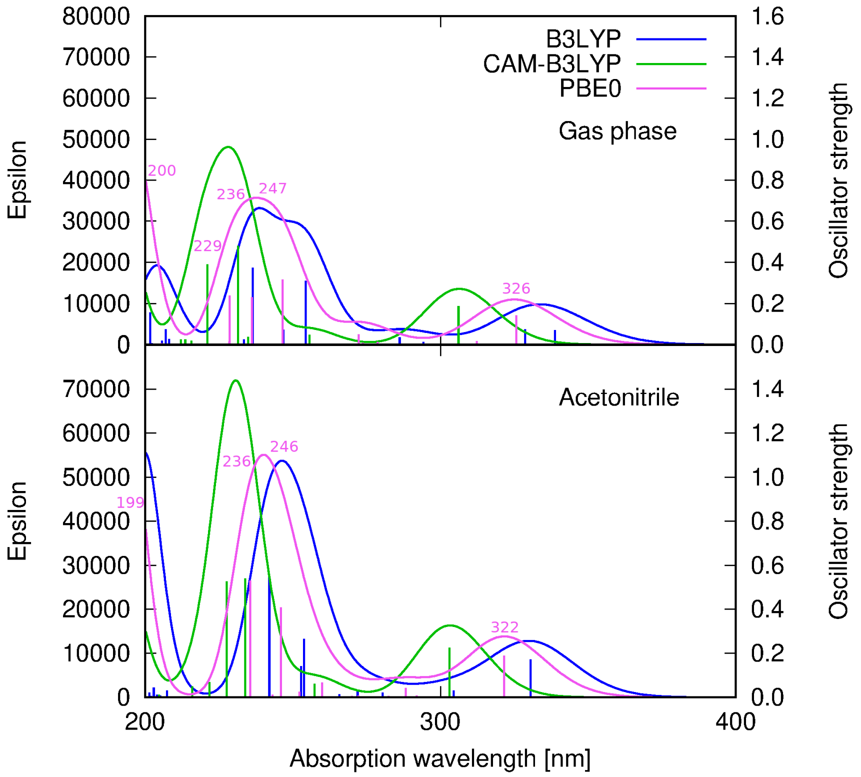

Figure S16. Theoretical prediction of the absorption spectrum of ligand **HL1** absorption spectrum in gas phase (upper panel) and acetonitrile (lower panel) calculated with B3LYP (blue line), CAM-B3LYP (green line) and PBE0 (violet line) functionals (numerical values of absorption wavelengths for most intensive transitions given for PBE0 functional).

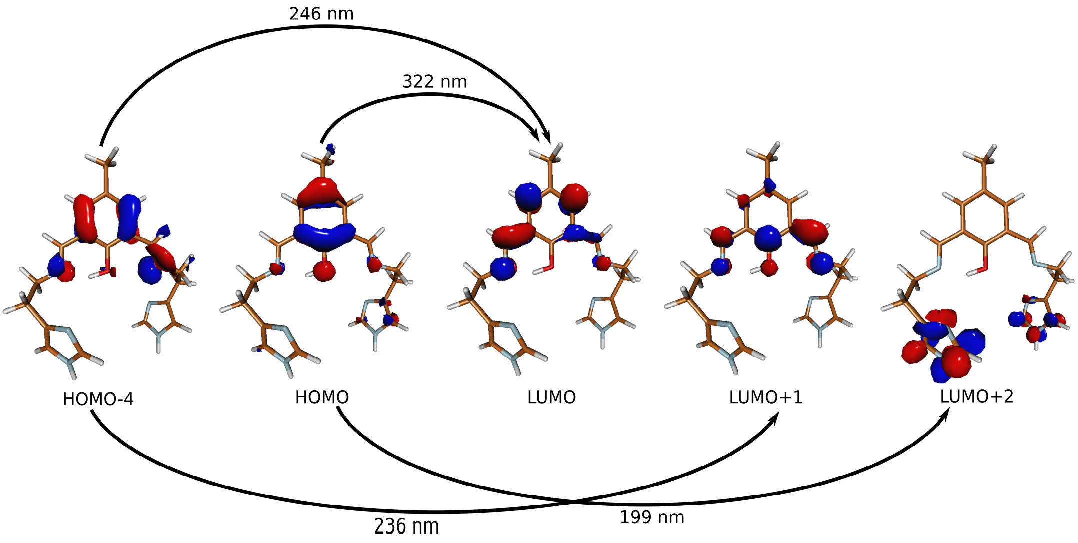

Figure S17. Frontier orbitals of ligand **HL1**, involved in most intenstive transitions (PBE0/def2-TZVP/acetonitrile) with corresponding wavelengths.

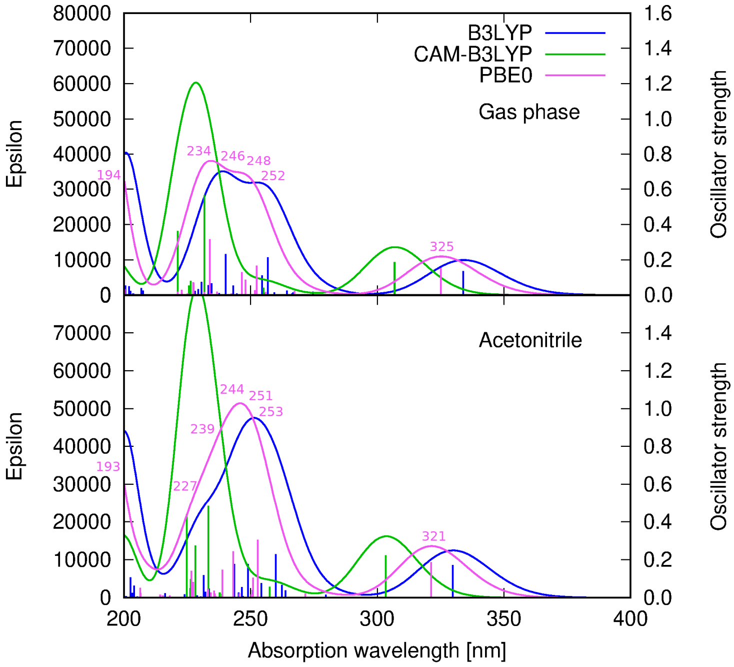

Figure S18. Theoretical prediction of the absorption spectrum of ligand **HL2** in gas phase (upper panel) and acetonitrile (lower panel) calculated with B3LYP (blue line), CAM-B3LYP (green line) and PBE0 (violet line) functionals (numerical values of absorption wavelengths for most intensive transitions given for PBE0 functional).

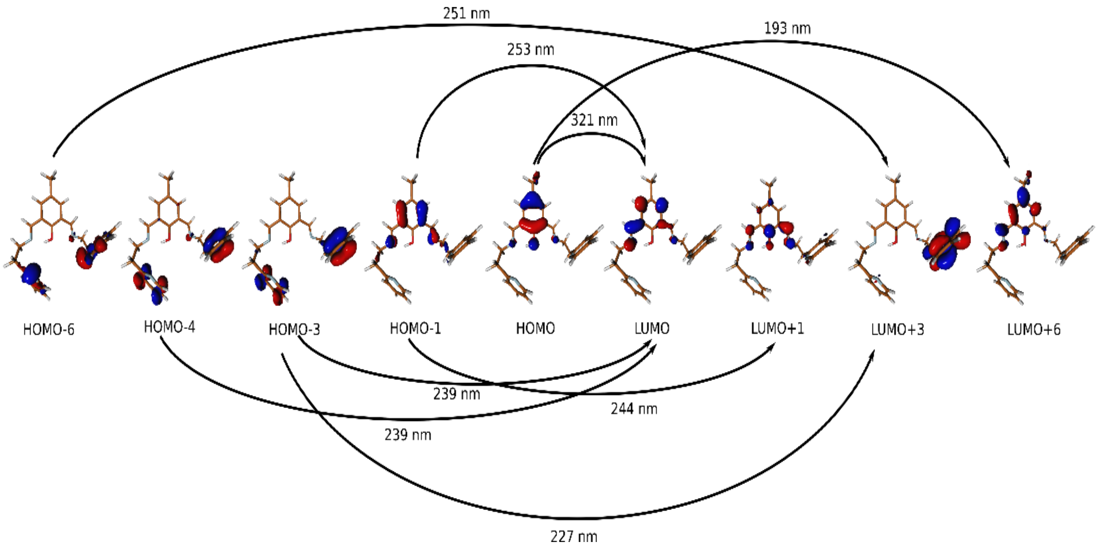

Figure 19. Frontier orbitals of ligand **HL2**, involved in most intenstive transitions (PBE0/def2-TZVP/acetonitrile) with corresponding wavelengths**.**

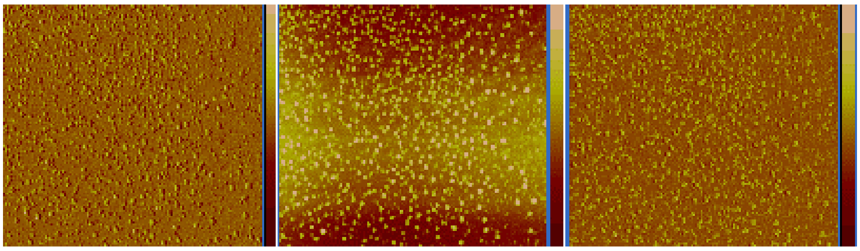

height amplitude phase

Figure S20. {[Cu_2(L1)Cl_2]$_2$[$CuCl_4$]}·2MeCN·2H_2O (**1**)/Si, scan size 5 μm, multistage spin coating. Phase images map of layer property including mechanical, chemical, and viscoelastic. Amplitude- the image of height, in which the dimension of *z* axis was reduced.

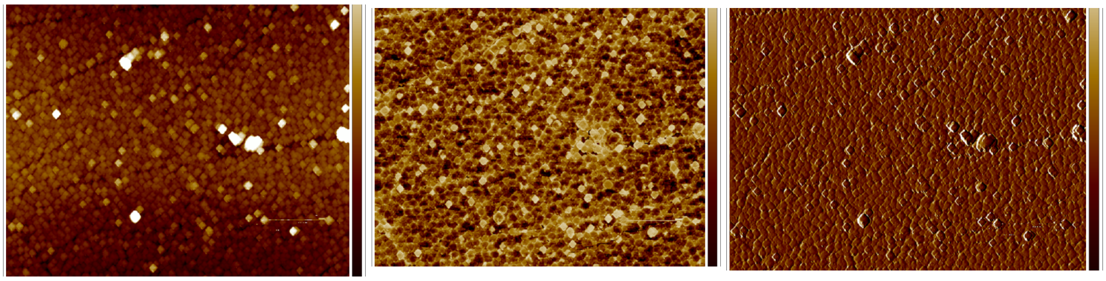

(a) (b) (c)

Figure S21. [Cu_2(L2)Cl_3] 0.5 MeCN·**2** /PMMA/Si a) height, b) phase, c) amplitude, scan size 5 μm, R_a = 5.15-19.9 nm, R_q = 3.66-25.9 nm, height 15 nm, 3000 rpm PMMA x1, complex **2** 3000 rpm x2, complex + PMMA 3000 rpm x1, complex 3000 rpm x1, 5s.

Literature

1. Graham, B.; Hearn, M.T.W.; Junk, P.C.; Kepert, C.M.; Mabbs, F.E.; Moubaraki, B.; Murray, K.S.; Spiccia, L. Syntheses, Crystal Structures, Magnetic Properties, and EPR Spectra of Tetranuclear Copper(II) Complexes Featuring Pairs of "Roof-Shaped" Cu2X2 Dimers with Hydroxide, Methoxide, and Azide Bridges. *Inorg. Chem.* **2001**, *40*, 1536-1543.
2. Chaudhuri, P.; Ventur, D.; Wieghardt, K.; Peters, E.M.; Simon, K.A. Preparation, Magnetism, and Crystal Structures of the Tautomers $[LCu(\mu_2\text{-}OH)_2CuL](ClO_4)_2$ (Blue) and $[LCu(\mu_2\text{-}OH_2)(\mu_2\text{-}O)CuL](ClO\mu_4)\mu_2$ (Green): μ-Aqua-μ-oxo vs. Di-μ-hydroxo Linkage. *Angew. Chem., Int. Ed. Engl.* **1985**, *24(1)*, 57-59.
3. Buchtelova, H.; Skubalova, Z.; Strmiska, V.; Michalek, P.; Kociova, S.; Smerkova, K.; Kruszyński, R.; Bieńko, A.; Kaj, M.; Lewińska, A.; Bieńko, D.; Malik-Gajewska, M.; Milosavljevic, V.; Kopel, P.; Heger, Z.; Adam, V. Synthesis and structural characterization of antimicrobial binuclear copper(II) coordination compounds bridged by hydroxy- and/or thiodipropionic acid. *J. of Inorg. Biochem.* **2019**, *191*, 8-20.
4. Charlot, M.F.; Jeannin, S.; Jeannin, Y.; Kahn, O.; Lucrece-Aubal, J.; Martin-Frere, J.S. Crystal structure and magnetic properties of tetrakis(cyclohexylamine)di-.mu.-hydroxo-dicopper(II) perchlorate. The first example of a roof-shaped hydroxo-bridged copper(II) dimer. *Inorg. Chem.* **1979**, *18(6)*, 1675-1681.
5. Charlot, M.F.; Kahn, O.; Jeannin, S.; Jeannin, Y. Exchange interaction in roof-shaped hydroxo-bridged copper(II) dimers. *Inorg. Chem.* **1980**, *19*, 1410-1411.
6. Farrugia, L.J.; Lovatt, P.A.; Peacock, R.P. Synthesis of a series of novel binucleating ligands based on 1,4,7-triazacyclononane and o-, m- and p-xylene: crystal structure of the μ-hydroxy-bridged dicopper(II) complex $[Cu_2L^m(OH)_2][BPh_4]_2$ $[L^m = \alpha,\alpha'$-bis (N-1,4,7-triazacyclononane)-m-xylene]. *J. Chem. Soc. Dalton Trans.* **1997**, 911-912.
7. Henry-Mowatt, J.; Dive, C.; Martinou, J.C.; James, D. Role of mitochondrial membrane permeabilization in apoptosis and cancer. *Oncogene* **2004**, *23*, 2850-2860.
8. Thakurta, S.; Roy, P.; Rosair, G.; Gómez-García, C.J.; Garribba, E.; Mitra, S. Ferromagnetic exchange coupling in a new bis(μ-chloro)-bridged copper(II) Schiff base complex: Synthesis, structure, magnetic properties and catalytic oxidation of cycloalkanes. *Polyhedron* **2009**, *28*, 695-702.
9. Bernalte-Garcia, A.; Lozano-Vila, A.M.; Luna-Giles, F.; Pedrero-Marin, R. Structural characterization of a thiazoline-pyrazole ligand and its complexes with cobalt(II) and copper(II). *Polyhedron* **2006**, *25*, 1399;
10. Connor, C.J.O. Ferromagnetic interactions in dimeric dichloro-[2,2-bis-(2-pyridyl)-1,3-dioxolane]copper(II). *Inorg. Chim. Acta* **1987**, *127*, L29-L30.
11. Tuna, F.; Patron, L.; Journaux, Y.; Andruh, M.; Plass, W.; Trombe, J.C. Synthesis and magnetic properties of a series of bi- and tri-nuclear complexes of copper(II) with the unsymmetrical tetradentate Schiff-base ligand 3-[N-2-(pyridylethyl)formimidoyl]salicylic acid, H2fsaaep, and crystal structures of $[\{Cu(Hfsaaep)Cl\}_2]$ and $[\{Cu(fsaaep)(H_2O)\}_2]$. *J. Chem. Soc., Dalton Trans.* **1999**, 539-544.
12. Sikdar, Y.; Modak, R.; Bose, D.; Banerjee, S.; Bieńko, D.; Zierkiewicz, W.; Bieńko, A.; Das Saha, K.; Goswami, S. Doubly chloro bridged dimeric copper(II) complex: magneto-structural correlation and anticancer activity. *Dalton Trans.* **2015**, *44*, 8876-8888.
13. Drake, R.F.; Crawford, V.H.; Laney, N.W.; Hatfield, W.E. Magnetic properties of di-.mu.-chlorobis[dichloro(guaninium)copper(II)] dihydrate. Second determination. Inorg. *Chem.* **1974**, *13(5)*, 1246-1249.
14. Carrabirie, J.A.; Sundaralingarn, M. Stereochemistry of Nucleic Acids and Their Constituents. VIII. Metal Binding Studies. Crystal Structure of a Guanine-Copper Chloride Complex, a Trigonal-Bipyramidally Coordinated Copper. *J. Am. Chem. Soc.* **1970**, *92(2)*, 369-371.
